# A systematic review and meta-analysis of breastfeeding and neurodevelopmental outcomes in preterm infant

**DOI:** 10.3389/fpubh.2024.1401250

**Published:** 2024-11-21

**Authors:** Ruolin Zhang, Erya Ying, Xiujuan Wu, Han Qin, Yanping Guo, Xin Guo, Zhangbin Yu, Jun Chen

**Affiliations:** ^1^Department of Neonatology, Nanshan Maternity & Child Healthcare Hospital, Shenzhen, Guangdong, China; ^2^Department of Pediatrics, Peking University Shenzhen Hospital, Shenzhen, Guangdong, China; ^3^Department of Neonatology, Longgang District Maternity & Child Healthcare Hospital of Shenzhen City, Longgang Maternity and Child Institute of Shantou University Medical College, Shenzhen, China; ^4^Department of Neonatology, Shenzhen People's Hospital, The Second Clinical Medical College of Jinan University, First Affiliated Hospital of Southern University of Science and Technology, Shenzhen, China

**Keywords:** preterm infant, breastfeeding, neurodevelopmental outcomes, cognitive, motor, neurodevelopmental impairment

## Abstract

**Background:**

Prematurity significantly impacts neonatal health worldwide, necessitating effective interventions to improve outcomes for these vulnerable infants. While breastfeeding has emerged as a cornerstone of preterm care, its precise impact on neurodevelopment remains a subject of ongoing inquiry and debate. This systematic review aims to investigate the existing evidence in this area.

**Methods:**

On December 17, 2023, online databases including PubMed, The Cochrane Library, Embase, Web of Science, CNKI, VIP, CBM, and Wan Fang Data were searched. Comparisons were classified into several categories: never breastfeeding (Never-BF) versus exclusive breastfeeding, Never-BF versus any breastfeeding (Any-BF), predominant preterm formula (Pre-PTF) versus predominant breastfeeding (Pre-BF), and Pre-PTF versus predominant donor breast milk (Pre-DBM) groups. Randomized controlled trials and observational studies were analyzed separately through meta-analyses. Each study’s risk of bias was assessed, and the GRADE system was utilized to evaluate the certainty of the findings.

**Results:**

Sixteen studies met the inclusion criteria, comprising one RCT and 15 cohort studies. The key findings indicated that infants in the Any-BF groups demonstrated superior long-term cognitive scores compared to those in the Never-BF groups, particularly evident in infants assessed before 18 months in the Pre-BF groups versus Pre-PTF groups. A reduced risk of neurodevelopmental impairment was also observed in preterm infants in the Any-BF groups. Evidence regarding the effect of breastfeeding on motor development was inconclusive, except for potential motor improvement in extremely low birth weight infants in the Any-BF groups. Neither exclusive breastfeeding nor pre-DBM exhibited clear superiority over Pre-PTF in terms of neurodevelopmental outcomes for preterm infants. Caution is warranted due to potential publication bias impacting the assessment of breastfeeding’s impact on motor skills.

**Conclusion:**

Our systematic review supports current recommendations for breastfeeding in preterm infants, emphasizing its positive effects on cognitive abilities and reduced risk of neurodevelopmental disorders. Further studies are needed to clarify if DHM provides neurodevelopmental benefits comparable to maternal milk, as current evidence does not sufficiently address this question. Additionally, future investigations should prioritize refining our understanding of the influence of breastfeeding on motor development in this vulnerable population.

**Systematic review registration:**

PROSPERO, identifier CRD42023492274, Available at: https://www.crd.york.ac.uk/prospero/display_record.php?ID=CRD42023492274.

## 1 Introduction

In 2020, approximately 13.4 million live births were premature, affecting almost 9.9% of global live births (1). This figure represents a staggering public health burden, as nearly 900,000 neonatal deaths result from direct complications of premature birth annually (2). Recent global data suggests that preterm births remain a pressing issue in countries like China, which contributes significantly to global numbers with 6.1% of its births being preterm, totaling more than three quarters of a million (3). Premature birth is not just an issue of early survival but one of long-term developmental health, contributing to neonatal mortality, as well as long-term adverse outcomes such as neurodevelopmental impairments, chronic diseases, and considerable economic costs (2–7). Given their vulnerability, preterm infants require special attention and intensified care (8). Among the most effective interventions available is breastfeeding.

Breastfeeding emerges as a crucial intervention for premature infants, providing well-established protection against complications associated with prematurity (8–10). It has a positive impact on both short-term and long-term outcomes, particularly in terms of neurodevelopment (11). The World Health Organization (WHO) and other international health organizations advocate for exclusive breastfeeding for the first 6 months of life, followed by continued breastfeeding alongside appropriate complementary feeding throughout the second year (12, 13). Additionally, China’s 2018 guidelines on Breastfeeding Promotion Strategies align with WHO recommendations, emphasizing the initiation of breastfeeding within the initial 30 min post-birth (14).

Breastfeeding confers protection against common diseases such as gastrointestinal tract infection, atopic eczema, respiratory infections, and otitis media (8). It also mitigates the risk of type 2 diabetes and childhood overweight/obesity (15). Furthermore, breastfeeding promotes cognitive (16–18) and motor skill (19, 20) development, which are critical indicators of neurodevelopmental outcomes (NDOs) in early life. Moreover, numerous studies show that breastfed children have higher verbal intelligence and, in boys, higher intelligence quotient (IQ) scores, total brain volume and white matter volume at 12 to 18 years of age, suggesting that the beneficial effects of breastfeeding continue from the NICU to adolescence (21–23). Evidently, breastfeeding positively influences the future health of the child, resulting in reduced healthcare burdens for health systems. However, despite international recommendations and documented benefits, global rates of exclusive breastfeeding for at least 6 months remain low at 40% (10, 24), and in China at 47.9% (25, 26), contrasting with WHO’s suggestions.

While ample evidence supports the association between breastfeeding and improved medical outcomes (27–29), whether breastfeeding exerts a lasting impact on children’s NDOs remains a contentious issue (30, 31). Several studies indicate a positive association between breastfeeding and NDOs (32–34). However, critics argue that enhanced NDOs are more closely linked to maternal education, socioeconomic status, IQ, and sensitivity rather than the nutrients in breast milk (35, 36). Thus, it is crucial to systematically review evidence both supporting and opposing the hypothesis that breastfeeding reduces the risk of suboptimal NDOs and determine if there is an independent reproducible effect of breastfeeding on NDOs in preterm infants. This systematic review and meta-analysis aim to synthesize the available evidence.

## 2 Materials and methods

### 2.1 Registration

Our systematic review and meta-analysis followed the PRISMA criteria (37) (Supplementary Table S1) and MOOSE guidelines (38) (Supplementary Table S2). Registration details for this review are documented in PROSPERO, the international prospective register of systematic reviews (Registration ID: CRD42023492274).

### 2.2 Criteria for selected studies for this review

This meta-analysis systematically assessed global studies exploring the correlation between breastfeeding and NDOs in preterm infants.

#### 2.2.1 Included studies met the following criteria

a) Participants: Preterm infants (gestational age < 37 weeks);
b) Interventions: Provision of breastfeeding during the infants’ hospital stay;
c) Comparisons: Evaluation of different feeding strategies (including exclusive, any, and never breastfeeding) and varying doses of breastfeeding administration;
d) Outcomes: NDOs. The primary outcome was cognitive scores measured in preterm infants at follow-up using validated assessment tools. Secondary outcomes included neurodevelopmental scores related to language and motor domains and the incidence of neurodevelopmental impairment (NDI), defined as the presence of one or more of the following: Bayley Scales of Infant Development (BSID-II/III) cognitive or motor scores below 70, Kaufman Assessment Battery for Children (KABC) mental processing scores below 85, blindness, deafness requiring hearing aids or cochlear implants, or cerebral palsy;
e) Study design: Randomized controlled trials (RCTs) and observational cohort studies, both prospective and retrospective.

#### 2.2.2 Exclusion criteria were applied in the following cases

a) Inclusion of term neonates;
b)Studies involving the same participants but with different aims;
c) Restriction of reported outcomes to behavioral/temperamental assessments or isolated motor skills, which are of uncertain value as prognosticators of long-term neurodevelopment or cognitive function;
d) Ineligible publication types, such as meta-analyses, systematic reviews, editorials, commentaries, guidelines, pilot studies, or case reports;
e) Failure to meet the designed inclusion criteria.

### 2.3 Intervention protocol

The intervention compared the effects of various breastfeeding-related feeding strategies. The comparison groups were categorized as follows:

a) Never Breastfeeding (Never-BF) versus Exclusive Breastfeeding (Exclusively BF);
b) Never-BF versus Any Breastfeeding (Any-BF);
c) Predominant Preterm Formula (Pre-PTF) versus Predominant Breastfeeding (Pre-BF);
d) Pre-PTF versus Predominant Donor Breast Milk (Pre-DBM).

### 2.4 Search strategy

Two researchers (RZ, EY) conducted a comprehensive search across databases, including PubMed, The Cochrane Library, Embase, Web of Science, CNKI, VIP, CBM, and Wan Fang Data, on December 17, 2023. Publications before January 2000 were excluded to ensure that the findings reflect current knowledge. The search was limited to studies published in English or Chinese. Additional references were sourced from cited literature. The search terms included “breast milk,” “human milk,” “breastfeeding,” and relevant MeSH terms, with neonate limiters specific to each database. Detailed search strategies are available in the Supplementary Table S3.

### 2.5 Literature retrieval and data extraction

Data extraction was performed by two independent authors (RZ, EY) using predesigned forms. In case of disagreement, a third author (XW) mediated discussions to reach a consensus. Extracted information included study details (authorship, publication year, location), population characteristics (sample size, gestational age, birth weight), and intervention, comparison, and outcomes.

### 2.6 Methodological quality evaluation

The Risk of Bias (RoB) tool from the Cochrane Collaboration was used to evaluate bias in RCTs. This tool consists of six domains: randomization method, allocation concealment, blinding, handling of incomplete outcome data, selective reporting, and other sources of bias (39). Additionally, the Newcastle-Ottawa Scale (NOS) (40) was used to assess and assign scores for the quality of cohort studies. The NOS assesses sample selection, cohort comparability, and outcome assessment. Sample selection is evaluated based on four criteria: the representativeness of the exposed cohort, the selection of the non-exposed cohort, the ascertainment of exposure, and the demonstration that the outcome of interest was absent at the study’s start. Cohort comparability, either through design or analysis, is also essential. Additionally, it is important to address factors such as outcome assessment, adequate follow-up for outcomes, and cohort follow-up adequacy. Quality assessment was conducted independently by two researchers (YG and XG), with any discrepancies resolved through arbitration by a third researcher (HQ) in cases of disagreement.

### 2.7 Statistical analysis

A meta-analysis was conducted to determine the correlation between breastfeeding and NDOs. Dichotomous outcomes were reported as relative risks (RR) with 95% confidence intervals (CI), while continuous variables were presented as standard mean differences (SMD) with corresponding 95% CI and underwent statistical analysis. The *I^2^* statistic was used to assess heterogeneity, categorized as not important (0 to 30%), moderate (30 to 50%), substantial (50 to 75%), or considerable (75 to 100%) (39), with corresponding *p*-values considered. If heterogeneity was present (*p* < 0.10 or *I^2^*≧50%), a random-effects model was used.

For studies reporting multiple statistical models, priority was given to the most adjusted one. When multiple studies provided data from the same sample, preference was accorded to the one with the most detailed results or the largest sample size. In cases of diverse neurodevelopmental assessment points, precedence was given to measurements the latest one.

The included studies, primarily prospective or retrospective observational studies, may present significant clinical or methodological variability. To address this, sensitivity analysis was conducted by removing one study at a time to evaluate its effect on the pooled SMD. Subgroup analyses were performed based on infants’ age at assessment (categorized as <18 months, 18–24 months, and > 24 months), gestational age (GA) and birth weight (BW) (GA <32 weeks and/or BW <1,500 g, and GA <28 weeks and/or BW <1,000 g), given the variability in GA and BW among the studies. Additional subgroup analyses considered whether studies adjusted for key confounders affecting NDOs, at least maternal education or IQ of covariates. To detect potential publication bias and the effect of small study sizes, funnel plots and Egger’s test were utilized (41). All statistical analyses were performed using RevMan 5.4.1 and Stata MP version 17.0 (Stata Corporation, College Station, TX).

### 2.8 Summary of findings and GRADE table

The Grading of Recommendations Assessment, Development, and Evaluation (GRADE) framework and GRADEPro guideline development tool software was used to create outcome-specific summary tables. These tables evaluated the quality of evidence, effect consistency, imprecision, indirectness, and publication bias for each outcome (42). High-quality evidence is considered to be RCTs with no limitations, while observational studies are considered to provide low-quality evidence. Studies can be downgraded by one (for serious concern) or two (for very serious concerns) based on the risk of bias, inconsistency, indirectness, imprecision, and publication bias. Observational studies with a large effect size have been upgraded by one for a strong association (43). For each outcome, we report our certainty in the findings as very low, low, moderate, or high, separately according to the study design (randomized controlled trials, observational studies).

## 3 Results

### 3.1 Literature screening process

**Figure 1:**
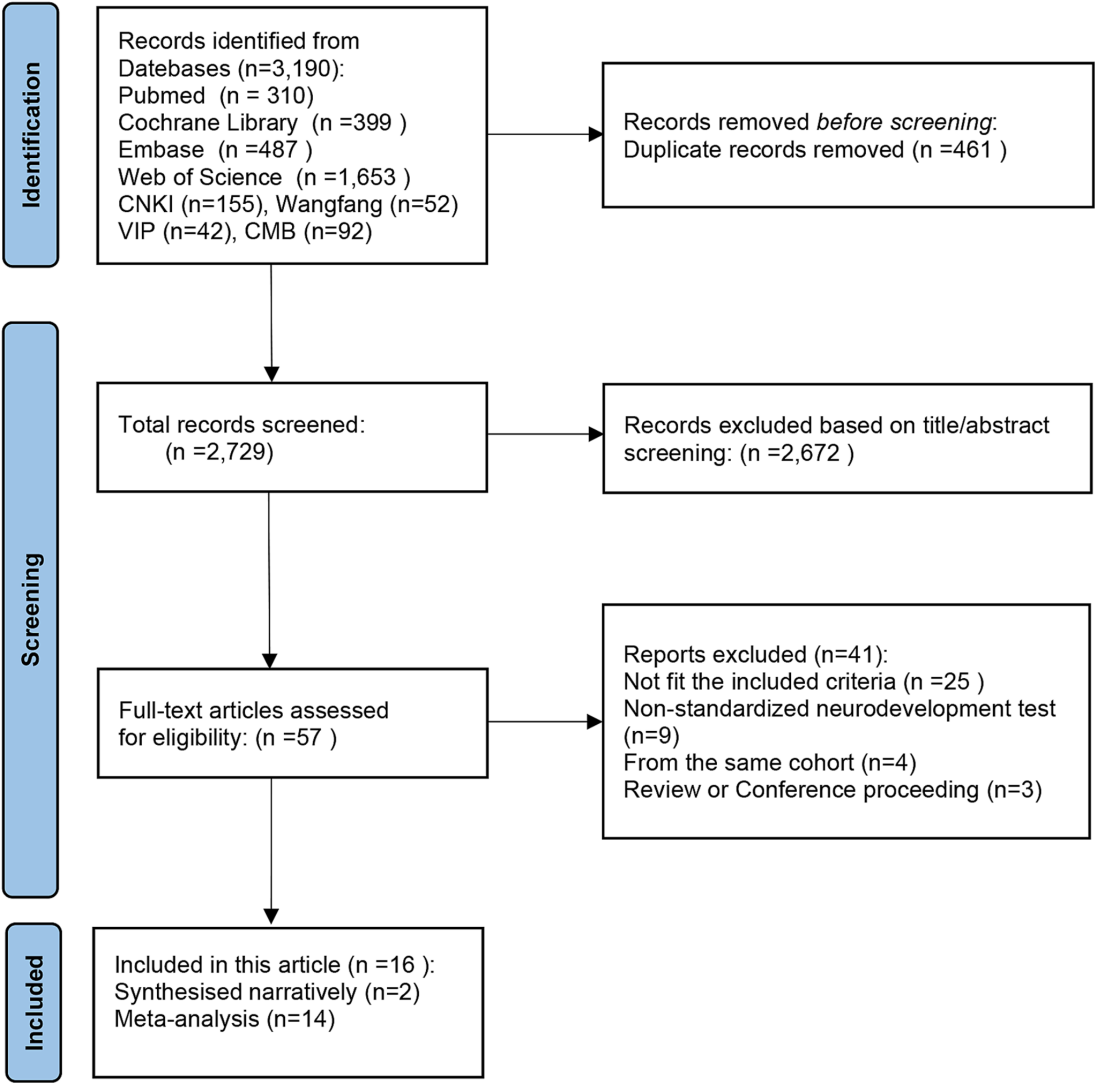
The PRISMA flow chart.

Systematically searching databases such as PubMed, The Cochrane Library, Embase, Web of Science, CNKI, VIP, CBM, and Wan Fang Data, a total of 3,190 studies were identified. Following the removal of 461 duplicates, the titles, and abstracts of the remaining 2,729 articles were reviewed. Employing predetermined criteria, 2,672 articles were excluded as they did not meet the inclusion criteria. After a thorough examination of the complete text, 41 articles were excluded for various reasons. Finally, 16 original studies were selected that met the criteria for systematic review and meta-analysis to ensure the reliability of the results. The retrieval procedures are illustrated in Figure 1 of the PRISMA flow chart (37).

The review examined data from 2000 to 2023, including 1 RCT and 15 cohort studies (66.7% prospective, 33.3% retrospective) from 8 different countries: Australia, Canada, Chile, Israel, Japan, Kenya, the UK, and the United States. For further details, please consult Table 1. Out of the selected studies (31, 36, 44–57), 14 were meta-analyzed (36, 44, 46–57), while 2 were synthesized narratively (31, 45) due to specific methodological differences or unavailable data for quantitative synthesis.

**Table 1:** Characteristics of studies included in the systematic review and meta-analysis.

Reference	Region	Study design	Enrolled duration	Sample size (*n*)	Inclusion criteria	Neurologic assessment	Outcomes	Assessment point	Adjustment for
Belfort et al. (2016) (31)	Australia	Prospective cohort study	2001 to 2003	180	GA < 30 wks., BW <1,250 g	BSID-II, MRI	MDI, PDI, IQ, brain volumes	24 months, 7 years	Maternal characteristics: age, education level, marital status, occupation, socioeconomic status, language. Neonatal characteristics: GA, gender, exposure to antenatal or postnatal corticosteroids, BPD (O_2_ @ 36 wk), NEC, sepsis
Bier et al. (2002) (57)	USA	Prospective cohort study	1996 to 1999	39	BW<2,000 g	BSID-II, AIMS	MDI, Motor development	3 months, 7 months, 12 months	Maternal characteristics: maternal PPVT score; Neonatal characteristics: No. of days of oxygen
Colacci et al. (2017) (56)	USA	Retrospective cohort study	2011 to 2013	85	GA < 37 wks., BW < 1,000 g	BSID-III	MDI, language, PDI, NDI	6 months, 12 months, 18 months	Maternal characteristics: education level, family income; Neonatal characteristics: GA, BPD, IVH, NEC, SAG
Feldman et al. (2003) (55)	Israel	Prospective cohort study	1996 to 1999	86	GA<33 wks., BW<1,750 g	BSID-II	PDI, MDI	6 months	Maternal characteristics: age, education level, marital status, socioeconomic status; Neonatal characteristics: GA, BW, the degree of medical risk, multiple births, apnea
Furman et al. (2004) (36)	USA	Prospective cohort study	1997 to 1999	119	GA < 33wk., BW 600–1,499 g	BSID-II, The Amiel-Tison	PDI, MDI, NDI	20 months	Maternal characteristics: education level, marital status, ethnicity. Neonatal characteristics: apnea, BPD (O2 @ 28 d), IVH, jaundice, NEC, PVL, sepsis
Hair et al. (2022) (54)	USA, Australia	Retrospective cohort study	2006 to 2010	252	BW ≤ 1,250 g	BSID-III	MDI, language, PDI	18–22 months	Neonatal characteristics: BW, gender, NEC, center
Jacobi-Polishook et al. (2016) (53)	Australia	Prospective cohort study	2001 to 2005	611	GA ≤ 33 wks.	BSID-II	PDI, MDI	18 months	*Maternal characteristics:* age, education level, smoking status during pregnancy, number of children and adults living at home, occupation, Home Screening Questionnaire score; *Neonatal characteristics:* GA, BW, gender, exposure to antenatal or postnatal corticosteroids, CRIB, IVH (3 to 4), NEC, ROP
Madore et al. (2017) (52)	USA	Prospective cohort study	2009 to 2012	81	BW < 1,000 g	BSID-III	MDI, language, PDI	12 months, 24 months	*Maternal characteristics:* social work involvement; *Neonatal characteristics:* multiple births, BPD
O’Connor et al. (2003) (50)	UK, USA, Chile	Retrospective cohort study	1996 to 1998	463	GA < 33wk., BW 750–1,800 g	BSID-II	PDI, MDI	12 months	*Maternal characteristics:* HOME Inventory, the vocabulary subtest of the maternal WAIS-R, smoking status during pregnancy and in-home; *Neonatal characteristics:* GA, SAG
O’Connor et al. (2016) (51)	Canada	RCT	2010 to 2012	363	BW<1,500 g	BSID-III	MDI, language, PDI	18 months	*Maternal characteristics:* education level; *Neonatal characteristics:* BW, dose of mother’s milk
Patra et al. (2017) (49)	USA	Retrospective cohort study	2008 to 2012	251	BW < 1,500 g	BSID-III	MDI, language, PDI	20 months	*Maternal characteristics:* education level, race/ethnicity; *Neonatal characteristics:* GA, gender, multiple births, apnea, BPD, NEC, SAG, sepsis, severe brain injury
Pinelli et al. (2003) (48)	Canada	Prospective cohort study	NR	148	BW <1,500 g	BSID-II	MDI, PDI	6 months, 12 months	*Maternal characteristics:* age, socioeconomic status; *Neonatal characteristics:* BW, dose of mother’s milk
Tanaka et al. (2009) (47)	Japan	Prospective cohort study	1999 to 2000	18	NR	KABC	MPC	5 years	NR
Vohr et al. (2006) (46)	USA	Prospective cohort study	1999 to 2001	1,035	BW < 1,000 g	BSID-II The Amiel-Tison	MDI, PDI, NDI	18–22 months	*Maternal characteristics:* age, education level, marital status, race, ethnicity; *Neonatal characteristics:* GA, gender, BPD (O_2_ @ 36 wk), IVH (3 to 4), NEC, PVL, SAG, sepsis
Were et al. (2006) (45)	Kenya	Prospective cohort	In 2002	120	BW < 1,000 g	Dorothy Egan’s Model, Saigaland Rosenbaum’s method	Developmental delay, Functional disability	24 months	NR
Yackobovitch-Gavan et al. (2023) (44)	Israel	Retrospective cohort study	2013 to 2015	131	GA < 32 wks.	The Griffiths Mental Development Scales	Locomotors; personal-social; hearing and language; eye and hand co-ordination and performance	12 months	*Maternal characteristics:* age, education level, socioeconomic status, type of pregnancy; *Neonatal characteristics:* GA, BW Z-score, gender, exposure to antenatal corticosteroids, multiple births, birth head circumference Z-score, apnea, mechanical ventilation

AIMS, The Alberta Infant Motor Scale; BSID, Bayley Scales of Infant Development; BF, breastfeeding; BPD, bronchopulmonary dysplasia; BW, birth weight; d, days; CLD, chronic lung disease; CRIB, clinical risk index for babies score; GA, gestational age; IVH, intraventricular hemorrhage; KABC, The Kaufman Assessment Battery for Children; MDI, The Mental Development Index; MPCs, The Mental Processing Composite Scale; NDI, neurodevelop-mental impairment; NR, not reported; PDI, The Psychomotor Development Index; PPVT, the peabody picture vocabulary test score; PVL, periventricular leukomalacia; ROP, retinopathy of prematurity; RCT, randomized control trial; SAG, small for gestational age; WAIS-R, The Revised Wechsler Adult Intel-ligence Scale; wks., weeks.

The study analyzed 16 selected studies conducted between 1996 and 2015, involving 3,982 preterm babies with sample sizes ranging from 18 to 1,035 participants. Seven studies (31, 36, 44, 48, 49, 51, 54) included infants born <32 weeks GA and/or < 1,500 g BW (*n* = 1,444). Additionally, four studies (45, 46, 52, 56) targeted infants born <28 weeks’ GA and/or with a BW < 1,000 g (*n* = 1,321). Five of the studies (47, 50, 53, 55, 57) focused on infants born <37 weeks’ GA and/or with a BW < 2,500 g, with a total of 1,199 participants. The studies analyzed in this research were conducted on preterm infants with varying GA and BW, and none of the study reported results stratified by GA and BW. Table 1 provides details of the included studies.

The studies by Were et al. (45) provided evidence for directly evaluating the advantages of Never-BF (where formula constituted the entire diet) versus exclusive BF (where breastfeeding constituted the entire diet, comprising mother’s own milk or DBM). Five articles (36, 44, 46, 53, 57) which included Never-BF, with exclusive BF or a combination of breastfeeding and formula. Eleven articles (31, 36, 47–50, 52–56) examined the effects of Pre-PTF, including low breastfeeding dose combined with preterm formula, compared to Pre-BF, including exclusive BF or predominant dose of breastfeeding combined with preterm formula. Three studies (51, 52, 54) provided DBM in either the intervention or control groups. The intervention in each study is described in detail in Table 2.

**Table 2:** Description of the intervention in selected studies.

Reference	Groups	Proportion of milk provided to the different groups			Fortifier	Comparisons for this review	Study duration	Scores of NOS
		Formula	MOM	DBM				
Belfort et al. (2016) (31)	NA	Undefined	>50% of nutrition	NA	Y	Pre-PTF vs. Pre-BF (Synthesized narratively)	First 4 wk. of life	9
Bier et al. (2002) (57)	Gp1	170 ± 258 mL/kg/wk.	852 ± 429 mL/kg/wk.	N/A	Y	Never-BF vs. Any-BF (Gp1 vs. Gp2)	Duration of hospitalization	7
	Gp2	100%	N/A	N/A				
Colacci et al. (2017) (56)	Gp1	NA	92% of times	Undefined	Y	Pre-PTF vs. Pre-BF (Gp1 vs. Gp2)	First 4 wk. of life	7
	Gp2	83 (17,100) % of times	Undefined	NA				
Feldman et al. (2003) (55)	Gp1	1,404.8 ± 602.7 mL	7,375.2 ± 3,211.6 mL (> 75% of nutrition)	N/A	NR	Pre-PTF vs. Pre-BF (Gp1 vs. Gp2)	Duration of hospitalization	7
	Gp2	7,848.1 ± 3,668.3 mL	970.2 ± 200.0 mL (< 25% of nutrition)	N/A				
Furman et al. (2004) (36)	Gp1	Undefined	≥50 mL/kg	N/A	Y	Never-BF vs. Any-BF (Gp1 + Gp2 + Gp3 vs. Gp4), Pre-PTF vs. Pre-BF (Gp1 vs. Gp2 + Gp3)	First 4 wk. of life	9
	Gp2	Undefined	25–49 mL/kg	N/A				
	Gp3	Undefined	1–25 mL/kg	N/A				
	Gp4	100%	N/A	N/A				
Hair et al. (2022) (54)	Gp1	NA	Undefined	Undefined	Y	Pre-PTF vs. Pre-BF (Gp1 vs. Gp2), Pre-DBM vs. Pre-PTF (Gp1 vs. Gp2)	From birth to 34–36 CA	7
	Gp2	Undefined	Undefined	NA				
Jacobi-Polishook et al. (2016) (53)	Gp1	Undefined	4th quartile of BM intake	N/A	Y	Never-BF vs. Any-BF (Gp1 + Gp2 + Gp3 + Gp4 vs. Gp5), Pre-PTF vs. Pre-BF (Gp1 + Gp2 vs. Gp3 + Gp4)	Duration of neonatal admission	8
	Gp2	Undefined	3rd quartile of BM intake	N/A				
	Gp3	Undefined	2nd quartile of BM intake	N/A				
	Gp4	Undefined	1st quartile of BM intake	N/A				
	Gp5	100%	NA	N/A				
Madore et al. (2017) (52)	Gp1	NA	100%	NA	Y	Pre-PTF vs. Pre-BF (Gp1 + Gp2 vs. Gp3), Pre-PTF vs. Pre-DBM (Gp2 vs. Gp3)	First month of life	6
	Gp2	NA	Undefined	>50%				
	Gp3	>50%	Undefined	NA				
O’Connor et al. (2003) (50)	Gp1	<100 mL/kg	>80%	N/A	Partial	Pre-PTF vs. Pre-BF (Gp1 + Gp2 vs. Gp3 + Gp4)	From initiation of enteral feeding to Term CA or hospital discharge	6
	Gp2	Undefined	≥50%	N/A				
	Gp3	Undefined	<50%	N/A				
	Gp4	>80%	<100 mL/kg	N/A				
O’Connor et al. (2016) (51)	Gp1	N/A	58.4% (13.6–96.0%) of nutrition	Undefined	Y	Pre-PTF vs. Pre-DBM (Gp1 vs. Gp2)	From consent to 90 d of life	N/A
	Gp2	Undefined	63.3% (9.6–97.2%) of nutrition	N/A				
Patra et al. (2017) (49)	Gp1	Undefined	132 ± 10(5th quintiles of BM intake)	NA	Y	Pre-PTF vs. Pre-BF (Gp1 + Gp2 + Gp3 vs. Gp4 + Gp5)	Duration of hospitalization	7
	Gp2	Undefined	103 ± 10(4th quintiles of BM intake)	NA				
	Gp3	Undefined	65 ± 16(3rd quintiles of BM intake)	NA				
	Gp4	Undefined	21 ± 7(2nd quintiles of BM intake)	NA				
	Gp5	Undefined	4 ± 4(1st quintiles of BM intake)	NA				
Pinelli et al. (2003) (48)	Gp1	Undefined	>80%	N/A	Y	Pre-PTF vs. Pre-BF (Gp1 vs. Gp2)	Duration of neonatal admission	7
	Gp2	Undefined	<80% or no	N/A				
Tanaka et al. (2009) (47)	Gp1	Undefined	>80%	N/A	NR	Pre-PTF vs. Pre-BF (Gp1 vs. Gp2)	Group allocation based on feeds within first month of life with outcome follow-up at 5 years	6
	Gp2	Undefined	<80%	N/A				
Vohr et al. (2006) (46)	Gp1	Undefined	Ranged from 1.0 to 110.6 mL/kg/d	N/A	Y	Never-BF vs. Any-BF (Gp1 vs. Gp2)	Duration of neonatal admission with 18-month outcome follow up for neurodevelopment	9
	Gp2	100%	N/A	N/A				
Were et al. (2006) (45)	Gp1	NA	100%	NA	NR	Never-BF vs. Exclusive-BF (Gp1 vs. Gp2) (Synthesized narratively)	Duration of neonatal admission	7
	Gp2	100%	NA	NA				
	Gp3	Undefined	Undefined	NA				
Yackobovitch-Gavan et al. (2023) (44)	Gp1	Undefined	134 (100, 149) mL/kg/d	NA	Y	Never-BF vs. Any-BF (Gp1 vs. Gp2)	First month of life	6
	Gp2	100%	NA	NA				

Data presented as mean ± SD or median (IQR) unless otherwise stated. BF, breastfeeding; CA, corrected Age; DBM, donor breast milk; Gp, group; NA, not applicable; NR, not reported; Pre-, predominant; wk., week; Y, yes.

### 3.2 Evaluation of bias in included studies

The examination of potential bias in RCT articles indicates a low risk, which can be ascribed to the proper implementation of randomization and blinding protocols. This ensures the objectivity of outcomes, thereby reducing the likelihood of bias. The evaluation of bias in cohort studies was conducted using the Newcastle-Ottawa Quality Assessment Scale (NOS), yielding scores ranging from 6 to 9 stars (Table 2). Eleven of them had a score of no less than 7, indicating that they were extremely high-quality studies with a low risk of bias. Further details on each item are provided in Supplementary Table S4.

### 3.3 Outcome assessment

The assessment periods varied from 6 months to 7 years. Most studies evaluated developmental outcomes during early childhood, specifically between 18 to 24 months (36, 45, 46, 49, 51–54, 56); however, five studies assessed outcomes prior to 18 months (44, 48, 50, 55, 57). In contrast, only two articles examined NDOs beyond 24 months to evaluate the long-term effects of NDOs in preterm infants (31, 47). For instance, Tanaka et al. investigated the association between breastfeeding—particularly the resulting DHA levels in the red blood cell membranes of infants—and the cognitive function of very low birth weight (VLBW) infants at 5 years of age (47). Similarly, a study conducted by Belfort et al. (31) focused on the effects of predominant breastfeeding on very preterm infants, measuring brain volumes and NDOs at 7 years of age.

The BSID emerged as the most prevalent outcome measure, with eight studies employing BSID-II (31, 36, 46, 48, 50, 53, 55, 57) and five utilizing BSID-III (49, 51, 52, 54, 56). Other assessments encompassed various developmental tests, including the Amiel-Tison (36, 46), the Kaufman Assessment Battery for Children (KABC) (47), the Alberta Infant Motor Scale (AIMs) (57), the Griffiths Development Scales (GMDS) (44), and assessments based on Dorothy Egan’s Model and Saigaland Rosenbaum’s method (45). In addition, the infants underwent the standard neurologic examinations listed in Table 1.

### 3.4 Never breastfeeding versus exclusive breastfeeding (*n* = 1)

Only one study, conducted by Were et al. (45), a prospective cohort investigation, focuses on the impact of exclusive BF on preterm infants born in Kenya weighing between 1,000 g and 1,500 g. The findings of this study indicate that neonates exclusively breastfed exhibit notably elevated rates of disability compared to those never exposed to breastfeeding at the age of 2 years (RR 2.04, 95% CI 1.1, 3.78, *p* = 0.02). However, it is imperative to underscore that the logistic regression analysis failed to ascertain early feeding as an independent prognosticator of functional disability. Additionally, the potential influence of confounding factors, such as maternal health and socio-economic status, was not controlled for, which may have affected the reliability of the study results.

Overall, this inquiry does not definitively demonstrate the superiority of Exclusively BF over Never-BF in improving the long-term NDOs of preterm infants.

### 3.5 Never breastfeeding versus any breastfeeding (*n* = 5)

Five cohort studies (36, 44, 46, 53, 57) have explored the impact of Never-BF versus Any-BF on NDOs in preterm infants born before 33 weeks’ gestation. Notably, four studies (36, 46, 53, 57) utilized the BSID-II assessment to evaluate cognition and motor at various ages (7, 12, 18, or 20 months), while 1 study employed the GMDS (44) to evaluate mental development at 12 months.

#### 3.5.1 Cognitive scores

Yackobovitch-Gavan et al. (44) reported that Any-BF in the first month was associated with a 6–7-point increase in GMDS scores at 12 months corrected age, adjusted for maternal factors, such as maternal age, education level, and socioeconomic status. Each 50 mL/kg/day increase in breastfeeding volume corresponded to a 2–3 point increase in GMDS scores (*p* < 0.01).

**Figure 2:**
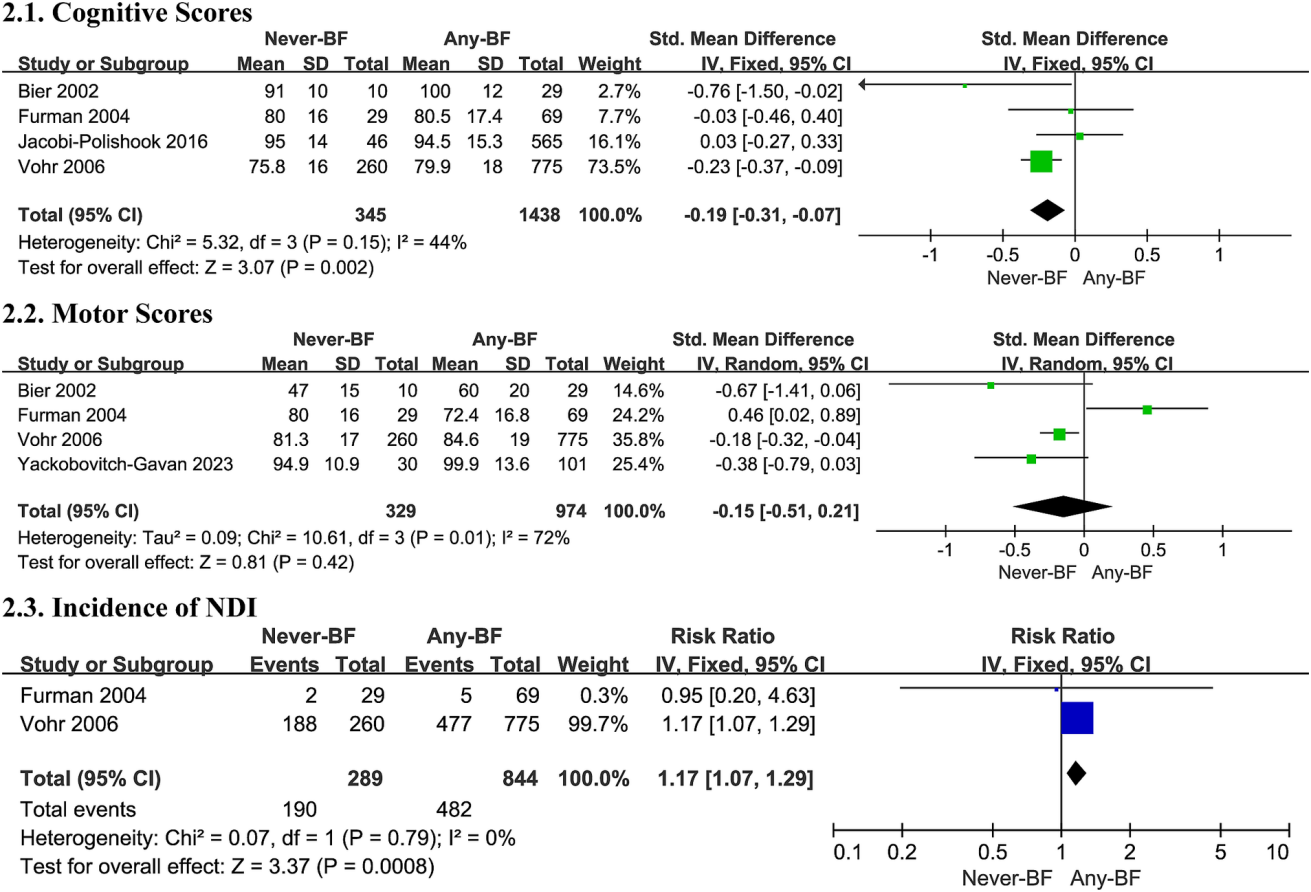
Effect size of Never-BF versus Any-BF. BF, breastfeeding; CI, confidence interval.

Four studies (36, 46, 53, 57) included in the meta-analysis found Any-BF associated with higher cognitive scores compared to Never-BF in a fixed effects model (SMD -0.19, 95% CI -0.31, −0.07, *p* = 0.002, Figure 2; very low certainty evidence; 4 observation studies, 1,783 participants, Supplementary Table S7), with non-significant evidence of heterogeneity (*I^2^* = 44%, *p* = 0.15).

#### 3.5.2 Motor scores

Five studies (36, 44, 46, 53, 57) contributed motor scores to the meta-analysis but failed to detect differences in motor development outcomes (SMD -0.08, 95% CI -0.37, 0.21, *p* = 0.60, Figure 2; very low certainty evidence; 5 observation studies, 1,303 participants, Supplementary Table S7), with a substantial heterogeneity in all studies (*p* = 0.007, *I^2^ =* 71%, Figure 2).

A random effects model and subgroup analysis revealed that infants born before 28 weeks of gestation or weighing less than 1,000 grams (SMD -0.18, 95% CI -0.04, −0.32, *p* = 0.01, Supplementary Table S6) and assessed prior to 18 months exhibited a slight advantage in motor scores. The observed heterogeneity may be attributed to variations in the populations studied and the differing time points at which NDOs were evaluated.

#### 3.5.3 Incidence of neurodevelopmental impairment

Two trials (36, 46) contributed data for a meta-analysis on the incidence of NDI, indicating a potential decrease in such impairment with any breastfed infants in a fixed effects model (RR 1.17, 95% CI 1.07, 1.29, *p* = 0.0008, Figure 2; very low certainty evidence; 2 observation studies, 1,133 participants, Supplementary Table S7), with no significant heterogeneity (*I^2^* = 0%, *p* = 0.79, Figure 2).

Overall, Any-BF is associated with enhanced cognitive outcomes and a possible reduction in NDI incidence in preterm infants, although evidence on motor development remains inconclusive.

### 3.6 Predominate preterm formula versus predominate breastfeeding (*n* = 11)

Eleven studies (31, 36, 47–50, 52–56) investigated the dose effect of breastfeeding on neurodevelopment, with 10 employing the BSID -II/III for cognitive, language, or motor assessment at various ages (6, 12, 18, 20, or 24 months), and 1 (47) using the KABC at 5 years of age. Ten cohort studies (36, 47–50, 52–56) were included in the meta-analysis. The study by Belfort et al. (31) was excluded from the meta-analysis because it primarily focused on the effect of breastfeeding on brain volume in very preterm infants at 7 years old and did not provide neurodevelopmental scores with means and standard deviations. However, the study reported a positive association between IQ and the number of days receiving >50% BF (0.5 points/day, 95% CI 0.2, 0.8).

#### 3.6.1 Cognitive scores

**Figure 3:**
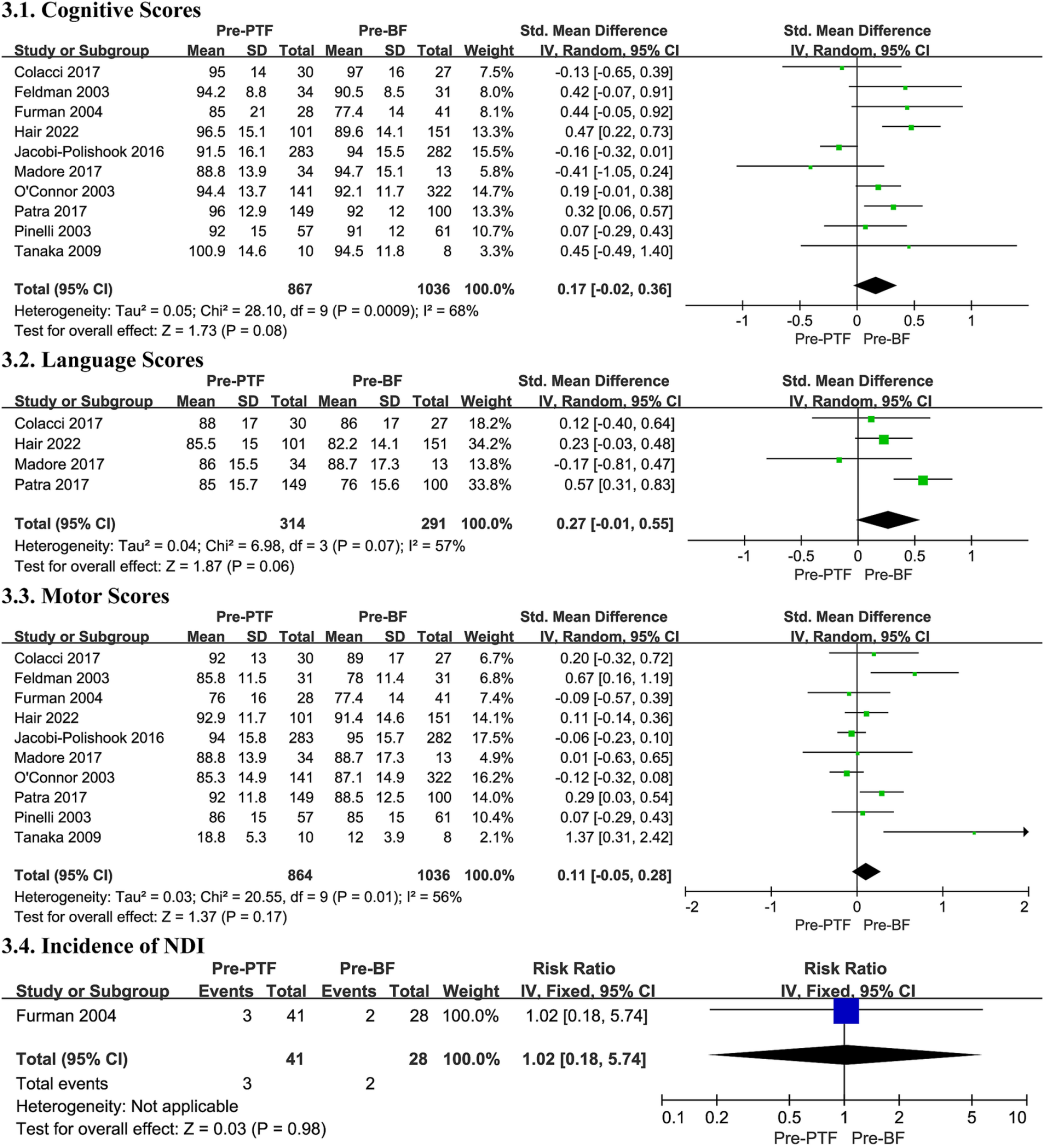
Effect size of Pre-PTF versus Pre-BF. Pre-, predominant; BF, breastfeeding; CI, confidence interval.

The meta-analysis, comprising 10 studies, found no statistically significant difference in cognitive outcomes between the Pre-PTF groups and the Pre-BF groups (SMD 0.17, 95% CI -0.02, 0.36, *p* = 0.08, Figure 3; low certainty evidence; 10 observation studies, 1,903 participants, Supplementary Table S7). However, substantial heterogeneity was present (*I^2^* = 68%, *p* = 0.0009, Figure 3), necessitating the use of a random effects model for evaluation and subsequent subgroup analysis.

Additionally, the subgroup analysis revealed a higher pooled SMD in cognitive scores for infants with less than 18 months of follow-up (SMD 0.19, 95% CI 0.03, 0.35, *p* = 0.02, Supplementary Table S6). The heterogeneity observed may be attributed to the disparate assessment points and the varying doses of breast milk consumed.

#### 3.6.2 Language scores

Four trials contributed data to the meta-analysis on language scores, showing no significant difference within a random effects model despite substantial heterogeneity (SMD 0.27, 95% CI -0.01, 0.55, *p* = 0.06, *I^2^* = 57%, Figure 3; very low certainty evidence; 4 observation studies, 605 participants, Supplementary Table S7).

Subgroup analysis revealed no meaningful differences based on GA, BW, or maternal education/IQ (see Supplementary Table S6). Heterogeneity may be due to the varying doses of breast milk.

#### 3.6.3 Motor scores

Ten studies contributed to motor score analysis and found no differences in motor development outcomes with a substantial heterogeneity (SMD 0.11, 95% CI -0.05, 0.28, *I^2^* = 56%, *p* = 0.17, Figure 3; very low certainty evidence; 10 observation studies, 1,900 participants, Supplementary Table S7), between the Pre-PTF groups and the Pre-BF groups. The same heterogeneity applies.

Subgroup analysis was conducted concerning different assessment points and infant characteristics due to potential biases. No significant differences were observed in the subgroups, except in the study by Tanaka et al. (47), which suggested that heightened breastfeeding levels in preterm infants during the neonatal period may notably influence brain development, especially in motor function at 5 years (SMD -1.37, 95% CI -2.42,-0.31, *p* = 0.01, Supplementary Table S6).

#### 3.6.4 Incidence of neurodevelopmental impairment

In the present meta-analysis, we included only 1 studies, which were conducted by Furman et al. (36) try to explore the correlation between the incidence of NDI and breastfeeding, to focusing on the impact of various breastfeeding dosages, and found no significant difference (RR 1.02, 95% CI 0.18, 5.74, *p* = 0.98, Figure 3; very low certainty evidence; 1 observation studies, 69 participants, Supplementary Table S7).

Overall, the evidence comparing Pre-PTF and Pre-BF on neurodevelopment is inconclusive, with no significant differences in cognitive, language, motor outcomes, or NDI.

### 3.7 Predominate preterm formula vs. predominate donor breast milk

Three studies (51, 52, 54) compared the NDOs of Pre-PTF versus Pre-DBM in preterm infants, including 1 RCT and 2 cohort studies. The BSID-III was utilized for evaluation of NDOs between 18 and 24 months of age in all studies. Given the potential for heterogeneity resulting from the use of disparate research designs, we conducted separate meta-analyses of RCT studies and observational studies.

#### 3.7.1 Cognitive scores

**Figure 4:**
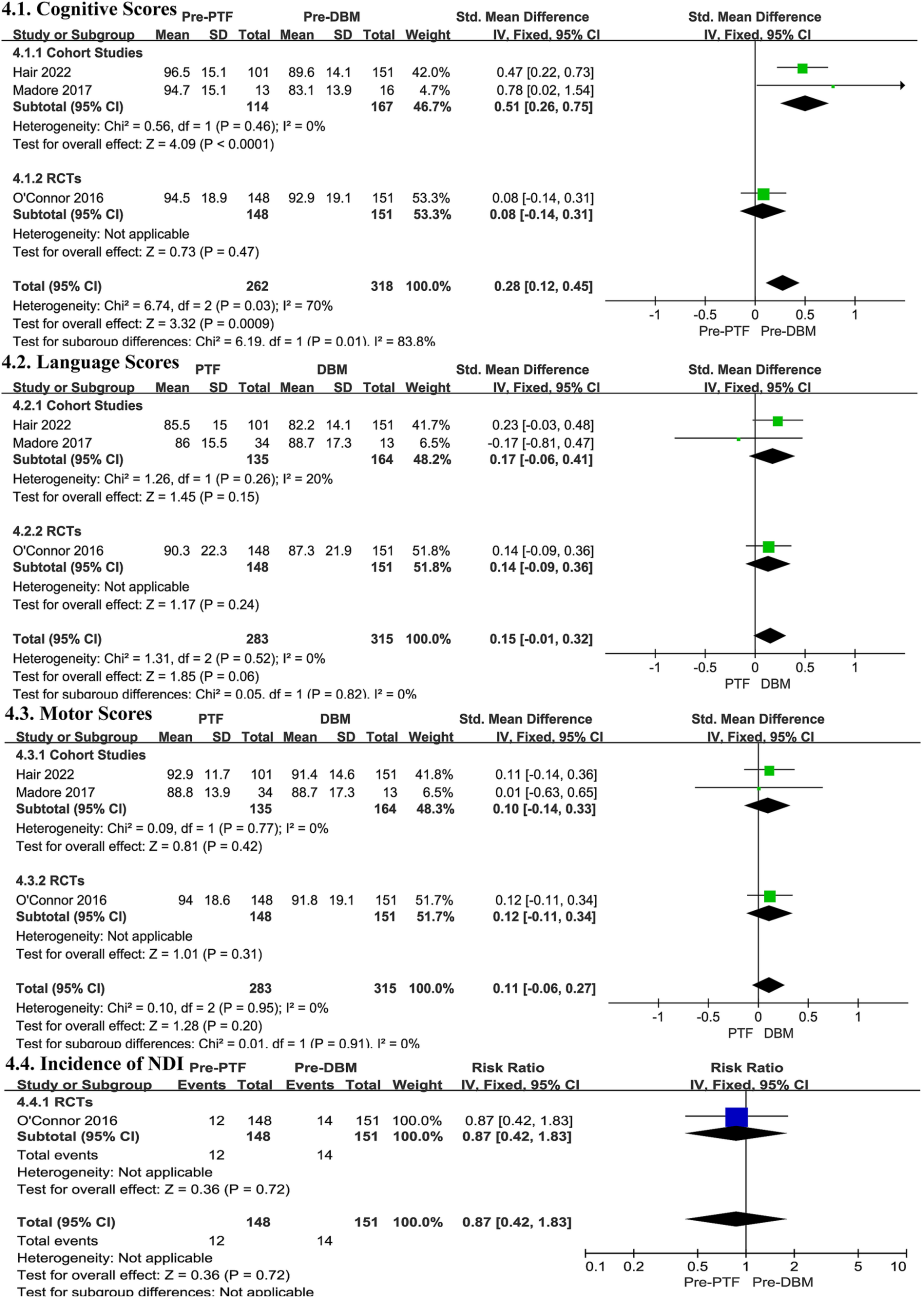
Effect size of Pre-PTF versus Pre-DBM. Pre-, predominant; DBM, donor breast milk; PTF, preterm formula; CI, confidence interval.

In the two cohort studies, the Pre-PTF group showed superior cognitive outcomes to the Pre-DBM groups (SMD 0.51, 95% CI 0.26, 0.75, *I^2^* = 0%, *p* < 0.0001, Figure 4; low certainty evidence; 2 observation studies, 281 participants, Supplementary Table S7), while the RCT did not find any significant differences (SMD 0.08, 95% CI -0.14, 0.31, *p* = 0.47, Figure 4; Moderate certainty evidence; 1 RCT, 299 participants, Supplementary Table S7). No heterogeneity was observed (Cohort studies: *I^2^* = 0%, *p* = 0.46; RCTs: Heterogeneity Not applicable; Figure 4).

#### 3.7.2 Language scores

In both cohort studies and RCT, the language outcomes for the Pre-DBM groups and Pre-PTF groups did not differ significantly (Cohort studies: SMD 0.17, 95% CI -0.06, 0.41, *p* = 0.15, Figure 4, low certainty evidence; 2 observation studies, 299 participants, Supplementary Table S7; RCTs: SMD 0.14, 95% CI -0.09, 0.36, *p* = 0.24, Figure 4, Moderate certainty evidence; 1 RCT, 299 participants, Supplementary Table S7), and there was no significant heterogeneity (Cohort studies: *I^2^* = 20%, *p* = 0.26; RCTs: Heterogeneity Not applicable; Figure 4).

#### 3.7.3 Motor scores

Similarly, in both cohort studies and RCT, motor scores did not exhibit any significant differences (Cohort studies: SMD 0.10, 95% CI -0.14, 0.33, *p* = 0.42, low certainty evidence; 2 observation studies, 299 participants, Supplementary Table S7; RCTs: SMD 0.12, 95% CI -0.11, 0.34, *p* = 0.31, Figure 4, Moderate certainty evidence; 1 RCT, 299 participants, Supplementary Table S7). The heterogeneity was not significant (Cohort studies: *I^2^* = 0%, *p* = 0.77; RCTs: Heterogeneity Not applicable; Figure 4).

#### 3.7.4 Incidence of neurodevelopmental impairment

Only the RCT (51) study provided data on the incidence of NDI, but no statistically significant differences were observed (RR 0.87, 95% CI 0.42, 1.83, *p* = 0.72, Heterogeneity Not applicable; Figure 4, Moderate certainty evidence; 1 RCT, 299 participants, Supplementary Table S7).

Overall, the evidence comparing Pre-PTF and Pre-DBM on cognitive function is inconclusive. No significant differences were observed in language, motor skills, or the incidence of NDI between the two feeding groups.

### 3.8 Publication bias and sensitivity analysis of the meta-analysis in observation studies

Supplementary Figures S1–S5 display funnel plots representing NDOs based on different feeding strategies. Publication bias was identified for the pooled effect sizes of motor development outcomes in preterm infants when comparing those fed Pre-PTF with those who received Pre-BF (Egger’s test: *p* = 0.048). This bias suggests a shortage of studies with larger sample sizes and effect sizes. Sensitivity analysis revealed that the pooled effect sizes shifted when individual studies were excluded. Specifically, the exclusion of Vohr et al. (46) altered the cognitive outcomes comparison between the Never-BF and Any-BF groups (effect size: -0.065, 95% CI: −0.299 to 0.169; Supplementary Table S5), indicating that this study significantly influenced the overall results. It is imperative to exercise caution when interpreting this finding, as heterogeneity and publication bias have the potential to impact its validity and generalizability.

## 4 Discussion

### 4.1 Summary of main findings

This systematic review and meta-analysis examined the association between breastfeeding and NDOs in preterm infants. The review encompassed studies from the past two decades, including 15 observational studies involving 3,619 preterm infants and one RCT with 363 preterm infants. Of these, 14 studies were included in the meta-analyses, while 2 studies involving 300 infants were synthesized narratively. We observed consistent evidence suggesting enhanced cognitive development in preterm infants who received any breastfeeding compared to those who were never breastfed, as well as a reduced risk of neurodevelopmental impairment. These findings reinforce existing recommendations for breastfeeding in preterm infants, highlighting its beneficial impact on cognitive abilities and the decreased likelihood of neurodevelopmental disorders. However, the effect of breastfeeding on motor development remains inconclusive. There is a slight positive effect of breastfeeding observed in infants born before 28 weeks of gestation or weighing less than 1,000 grams, indicating a need for further investigation. Additionally, the comparative superiority of exclusive breastfeeding, DBM, and various doses of breastfeeding versus PTF concerning long-term NDOs in preterm infants remains inconclusive. The variability in demographic characteristics, testing times, and the diversity of assessments employed further complicated the analysis of this outcome. Furthermore, the overall quality of the evidence for most results was assessed as low to very low, which significantly limits the credibility of the findings.

### 4.2 Strengths and weaknesses

A robust search strategy across eight electronic databases, adhering to Meta-analysis of Observational Studies in Epidemiology (MOOSE) guidelines, provided a comprehensive review of both RCTs and cohort studies, relevant to neonatal clinicians and parental decision-making, particularly concerning DBM. The high rate of premature births in China raises significant public health concerns, as preterm infants frequently face complications such as bronchopulmonary dysplasia (BPD), intraventricular hemorrhage (IVH), necrotizing enterocolitis (NEC), and long-term NDOs (58). While the prevalence of preterm births in China highlights the need for research on breastfeeding and NDOs, the lack of local studies presents an important area for future exploration.

The review did not encompass an evaluation of the effects of fortifiers; consequently, the existing evidence is insufficient to draw definitive conclusions regarding their impact on NDOs in preterm infants. Additionally, our search was confined to literature published in English and Chinese, which may have led to the omission of relevant studies. Furthermore, while we intended to stratify the analyses based on varying income settings, we identified no studies from low-income countries and only one (45) from a middle-income country. This limited geographic and economic representation has restricted our ability to make meaningful subgroup comparisons across income levels. Consequently, the findings presented here primarily represent contemporary practices in neonatal intensive care units (NICUs), as the majority of studies included in the meta-analysis originated from developed nations.

Simultaneously, we evaluated the quality of the included studies and found that most were observational, with only one RCT. As a result, the overall quality of evidence was generally weak. Methodological limitations were prevalent, including insufficient control of confounding variables (45, 47), small sample sizes (47, 52, 55), selection bias (44, 47, 49, 50, 54, 56, 57), and high rates of attrition (45, 47, 52). Many studies recruited infants born before 2010 (31, 36, 45–55, 57), and only two reported outcomes beyond 24 months (31, 47), making it difficult to assess the long-term effects of breastfeeding on NDOs in preterm infants. Additionally, some studies included infants with a GA of 32 weeks or more and/or a BW above 1,500 g (47, 50, 53, 55–57). None of these studies reported results stratified by GA and BW; therefore, the findings cannot be generalized to all preterm infants, particularly the very preterm infants (<1,500 g), who represent a more vulnerable subset of our population of interest.

Sources of bias in any meta-analysis include the selection and heterogeneity of the included studies. A specific limitation of our systematic review and meta-analysis concerns the date selection. Although our search criteria excluded studies published before 2000, some included studies relied on retrospective data from before 2000, potentially reflecting outdated practices and contributing to heterogeneity. This temporal variation complicates the synthesis of findings and may impact the applicability of conclusions to current clinical practices. While our inclusion criteria specified our population and outcomes of interest, we encountered heterogeneity, with some studies choosing to study only very preterm infants (<1,000 g or < 1,500 g), a more vulnerable subset of our population of interest, which may limit applicability. Additionally, combining studies with endpoint classifications ranging from 6 months to 7 years was challenging, with 58.8% of assessments conducted at 18 to 24 months. The absence of standardized endpoints for evaluating NDOs in preterm infants further complicated direct comparisons. Furthermore, while most studies reported breastfeeding rates, the methods used to quantify milk intake (e.g., “higher” vs. “lower”) differed significantly, particularly when comparing groups such as “Any-BF” vs. “Never-BF” or “Pre-BF” vs. “Pre-PTF.” The range of breastfeeding exposure varied from exclusive breastfeeding to as little as 9.6% of total intake, or it was inconsistently measured across studies. Given this variability, there is a pressing need for standardized neurodevelopmental assessment tools with long-term predictive validity, as well as further research into the complex relationship between breastfeeding and NDOs in preterm infants, based on more precise and consistent measurements of breastfeeding intake.

The majority of studies included in this review employed a rigorous approach to control for potential confounding variables, including neonatal complications, parental IQ, and socioeconomic differences. However, two studies lacked sufficient detail in defining these factors (45, 47). The methods employed to adjust for these covariates exhibited considerable variation across the included studies. The majority of the evidence reviewed is derived from observational studies, with only one randomized controlled trial (RCT) included, which may introduce a risk of bias. The objective of this review was to provide a comprehensive synthesis of the available evidence, and thus both RCTs and observational studies were included. The GRADE system was utilized to evaluate the certainty of the evidence, incorporating study design into the assessment. However, a considerable number of the studies had relatively small sample sizes, which complicated the interpretation of the evidence, particularly when comparing data from both RCTs and observational studies, where the observed effects may not align. To address potential sources of bias, we conducted subgroup analyses based on GA, BW, assessment time points, and maternal education/IQ, utilizing random-effects models to account for variability. A funnel plot and Egger’s test were employed to detect potential publication bias. The results indicated the presence of bias in one analysis, which resulted in a downgrade in the certainty of the evidence. This highlights the need for high-quality, large-scale, long-term studies that stratify results by GA and BW to more accurately investigate the relationship between breastfeeding and NDOs in preterm infants.

### 4.3 Mechanisms of the association between BF and NDOs

Breastfeeding plays a crucial role in the long-term neurodevelopment of preterm infants, with its benefits supported by evidence across multiple mechanisms. One critical aspect is the enhancement of maternal bonding through skin-to-skin contact and breastfeeding (59–62). This bonding not only strengthens the mother-infant attachment but also fosters emotional and cognitive development (63), often linked to increased maternal sensitivity and engagement in cognitively stimulating activities such as verbal interactions and sensory play (62). Furthermore, the nutritional composition of human milk is indispensable for both physical and neurological development in preterm infants (64). Rich in essential nutrients, human milk contains long-chain polyunsaturated fatty acids (PUFAs) (18, 65), particularly DHA, which are vital for brain development, contributing to synaptogenesis and myelination—critical processes in cognitive and visual development (66, 67). Moreover, the role of human milk oligosaccharides (HMOs) in neurodevelopment cannot be understated (68). HMOs foster a healthy gut microbiome, which in turn influences the gut-brain axis—an area of increasing interest in neuroscience. This connection highlights the interplay between nutrition and neurological development, suggesting that both the physical components of breast milk and the behavioral interactions surrounding breastfeeding are critical. Additionally, recent studies underscore the importance of immunological factors in breast milk, particularly immunoglobulin A (IgA), which protects the developing brain from neuroinflammation, a crucial element in preventing cognitive impairments (69). In summary, the synergistic effects of these components—enhanced maternal bonding, optimal nutrition, and immune protection—are especially critical during the brain’s periods of plasticity. During these critical periods, the combined influence of these factors can have lasting positive impacts on neurodevelopment, underscoring the importance of breastfeeding in the early stages of life.

### 4.4 Findings from other reviews

A previous systematic review evaluated eight observational studies involving 1,560 preterm infants and provided very low-certainty evidence regarding the impact of formula feeding versus breastfeeding NDOs (70). It concluded that breastfeeding had limited or no significant impact on cognitive and language neurodevelopment compared to formula feeding. Our updated review, which incorporated eight additional studies and expanded the participant pool to 3,982 infants, revealed altered effects on cognitive outcomes as of 2022 (70). However, the impact on motor and language development remained unchanged, and the overall certainty of the evidence did not improve.

Two recent Cochrane reviews (71, 72) and three non-Cochrane reviews (20, 73, 74) have further explored the impact of breastfeeding on NDOs in preterm infants. For instance, Brown et al. (72) conducted a Cochrane review but identified no relevant randomized controlled trials (RCTs) comparing the developmental outcomes between formula-fed and breastfed infants. Similarly, our review, despite utilizing a comprehensive search strategy, identified only one cohort study conducted by Were et al. (45), which suggested that extremely low birth weight (ELBW) infants fed formula exhibited better developmental outcomes at two years of age compared to those exclusively breastfed. However, the authors noted that NDOs in ELBW infants could be influenced by uncontrolled confounders, such as maternal health and socioeconomic factors. Furthermore, the study did not specify whether breast milk was routinely fortified, raising the possibility that inadequate nutrient intake from unfortified breast milk may have contributed to these findings. Research demonstrates that fortified breast milk can result in developmental outcomes comparable to those seen with preterm formula (75–77). This supports the hypothesis that insufficient nutrient supply disproportionately affects the most vulnerable infants—those with the highest nutritional demands—during the critical period of neonatal hospitalization (78).

A separate Cochrane review from 2019 provided moderate-certainty evidence that formula feeding promotes better weight gain and growth compared to DBM (71). However, these trial data did not indicate any long-term neurodevelopmental benefits. It is important to note that some studies included in these reviews focused on NDOs from the 1990s, making their relevance to contemporary clinical practice limited. As clinical protocols have advanced, the generalizability of these findings has decreased, leading to the exclusion of older studies from our review. Two cohort studies were identified: a retrospective cohort study (*n* = 252) (54) and a prospective cohort study (*n* = 81) (52). Both studies found cognitive improvements among preterm infants receiving donor breast milk; however, no significant effects were observed in language development, motor skills, or neurological function. In contrast, a randomized controlled trial by O’Connor et al. (51), consistent with Quigley et al. (71) review, found no benefits of donor breast milk in these outcomes for preterm infants. Thus, if donor milk is used in a setting with high maternal breast milk provision, it should not be regarded as a standalone intervention for improving NDOs. Several factors may explain the lack of observed improvements. Firstly, existing literature suggests a dose-dependent relationship between breastfeeding volume and NDOs in VLBW infants (46, 50, 79). While the studies included in our analysis adjusted for breast milk intake, it remains possible that the level of supplementation was insufficient to yield an observable effect on NDOs. Secondly, maternal breast milk and donor milk differ in nutrient and bioactive component composition (80). Pasteurization of donor milk may alter key bioactive components, potentially affecting neurodevelopment (80, 81). Lastly, donor milk is typically term milk, which may not meet the specific nutritional needs of preterm infants.

In addition to the Cochrane reviews, several non-Cochrane reviews have examined various outcomes, including in-hospital growth and neurodevelopment. A 2017 narrative review (74) suggested a modest protective effect of breastfeeding on neurodevelopment, while acknowledging numerous confounding variables such as neonatal complications, parental IQ, and socioeconomic status. Our current review re-examined six high-quality studies from this review and found that most had adjusted for major confounders, with only two exceptions (45, 47). However, it is important to recognize that confounding variables were not consistently controlled for across studies, which introduces the risk of publication bias and heterogeneity. To address this, we conducted subgroup analyses based on whether the key confounder—maternal education level or IQ—was controlled for, and found no significant impact on the primary outcomes. Consequently, promoting and supporting breastfeeding remains a critical public health goal. A meta-analysis by Miller et al. (73) sought to differentiate the effects of breastfeeding and preterm formula on NDOs but found insufficient evidence to draw definitive conclusions on cognitive and motor development. Our updated analysis, which incorporates more recent studies, consistently shows that any breastfeeding is associated with improved cognitive development and reduced neurodevelopmental impairment compared to exclusive formula feeding. However, no significant improvements were observed in predominantly breastfed preterm infants compared to those fed formula, which is consistent with the findings of Miller et al. (73). This divergence from conventional understanding may be attributable to differences in study populations, variations in breastfeeding intake and assessment points, and methodological inconsistencies across the studies included in our review. These findings underscore the need for more high-quality research, especially randomized controlled trials or well-designed cohort studies, to control for confounders and provide more reliable evidence.

Furthermore, a 2022 review by Hernandez-Luengo et al. focused on motor development and found that infants who were exclusively or ever breastfed had superior motor development compared to those never breastfed (20). These findings align with some outcomes in our review, suggesting that any breastfeeding positively impacts motor development in ELBW infants. However, it is crucial to acknowledge that the study conducted by Hernandez-Luengo et al. examined the influence of breast milk on motor assessments in individuals under the age of 18 years. Consequently, their findings may not entirely align with those of Miller et al.’s previous analysis and our results, which focused on a distinct group of preterm infants (73). Further research is necessary to validate whether breastfeeding during the neonatal period enhances long-term motor developmental outcomes in preterm infants, and to explore the effects of variables such as GA, BW, and breast milk fortification.

### 4.5 Implications for practice

This systematic review and meta-analysis provide robust support for current breastfeeding recommendations and underscore the critical role of feeding practices in enhancing NDOs among preterm infants. Given the significant cognitive and behavioral challenges commonly experienced by this population, the promotion of breastfeeding is of paramount importance. Notably, our analysis demonstrates a clear association between reduced breastfeeding rates and an elevated risk of suboptimal neurodevelopment, a relationship that is increasingly concerning in the context of rising preterm birth rates. As breastfeeding represents a modifiable factor, it is essential to educate parents on its neurodevelopmental benefits and to actively promote strategies that encourage increased breastfeeding rates, which may serve to mitigate the associated risks. Furthermore, in clinical settings where maternal breast milk is available in sufficient quantities, DHM should be regarded as a complementary intervention rather than a primary or standalone strategy for optimizing NDOs.

### 4.6 Implications for research

Further epidemiological research is critical to elucidate the relationship between breastfeeding and NDOs in preterm infants. Numerous confounding factors, including maternal age, education level, socioeconomic status, and the home environment, exert a significant influence on NDOs. However, inconsistent control of these variables across studies has contributed to a lack of consensus in the current body of evidence. Given the ethical challenges that limit the feasibility of conducting randomized controlled trials on breastfeeding, it is imperative to rigorously control for confounders and employ validated assessment tools to ensure the reliability and validity of findings in long-term prospective cohort studies conducted in real-world settings. This approach is particularly important in regions with elevated rates of preterm births, such as China. Addressing these methodological complexities will be a central focus of our forthcoming research.

## 5 Conclusion

In conclusion, this study advances the understanding of breastfeeding’s impact on NDOs in preterm infants. Despite inconsistencies in existing research, our findings indicate that breastfeeding, even in small amounts, may positively influence cognitive development in this population. Further studies are needed to clarify if DHM shows similar neurodevelopmental benefits, as these studies were not powered to study this outcome adequately. The certainty of the evidence was low, and the small effect sizes limit the clinical relevance of the findings. Further research is needed to confirm these results and explore the underlying mechanisms. This study underscores the importance of promoting breastfeeding to optimize NDOs in preterm infants.

## Data Availability

The original contributions presented in the study are included in the article/Supplementary material, further inquiries can be directed to the corresponding authors.
