# Supplementary material for: A systematic review and meta-analysis of breastfeeding and neurodevelopmental outcomes in preterm infant
Source: Front Public Health. 2024 Nov 21;12:1401250. doi: 10.3389/fpubh.2024.1401250 (PMC11617369; doi:10.3389/fpubh.2024.1401250)
Supplement: Supplementary file 1 [file Data_Sheet_1.pdf]

Supplementary Material

Table S1. PRISMA Checklist for this systematic review and meta-analysis.

Topic	No.	Item	Location where item is reported
**TITLE**			
Title	1	Identify the report as a systematic review.	1
**ABSTRACT**			
Abstract	2	See the PRISMA 2020 for Abstracts checklist	
**INTRODUCTION**			
Rationale	3	Describe the rationale for the review in the context of existing knowledge.	1, 2
Objectives	4	Provide an explicit statement of the objective(s) or question(s) the review addresses.	1, 2
**METHODS**			
Eligibility criteria	5	Specify the inclusion and exclusion criteria for the review and how studies were grouped for the syntheses.	2, 3
Information sources	6	Specify all databases, registers, websites, organisations, reference lists and other sources searched or consulted to identify studies. Specify the date when each source was last searched or consulted.	3
Search strategy	7	Present the full search strategies for all databases, registers and websites, including any filters and limits used.	3
Selection process	8	Specify the methods used to decide whether a study met the inclusion criteria of the review, including how many reviewers screened each record and each report retrieved, whether they worked independently, and if applicable, details of automation tools used in the process.	3
Data collection process	9	Specify the methods used to collect data from reports, including how many reviewers collected data from each report, whether they worked independently, any processes for obtaining or confirming data from study investigators, and if applicable, details of automation tools used in the process.	3
Data items	10a	List and define all outcomes for which data were sought. Specify whether all results that were compatible with each outcome domain in each study were sought (e.g. for all measures, time points, analyses), and if not, the methods used to decide which results to collect.	3
	10b	List and define all other variables for which data were sought (e.g. participant and intervention characteristics, funding	3, 4

		sources). Describe any assumptions made about any missing or unclear information.	
Study risk of bias assessment	11	Specify the methods used to assess risk of bias in the included studies, including details of the tool(s) used, how many reviewers assessed each study and whether they worked independently, and if applicable, details of automation tools used in the process.	3
Effect measures	12	Specify for each outcome the effect measure(s) (e.g. risk ratio, mean difference) used in the synthesis or presentation of results.	3, 4
Synthesis methods	13a	Describe the processes used to decide which studies were eligible for each synthesis (e.g. tabulating the study intervention characteristics and comparing against the planned groups for each synthesis (item 5)).	3, 4
	13b	Describe any methods required to prepare the data for presentation or synthesis, such as handling of missing summary statistics, or data conversions.	3, 4
	13c	Describe any methods used to tabulate or visually display results of individual studies and syntheses.	3, 4
	13d	Describe any methods used to synthesize results and provide a rationale for the choice(s). If meta-analysis was performed, describe the model(s), method(s) to identify the presence and extent of statistical heterogeneity, and software package(s) used.	3, 4
	13e	Describe any methods used to explore possible causes of heterogeneity among study results (e.g. subgroup analysis, meta-regression).	3, 4
	13f	Describe any sensitivity analyses conducted to assess robustness of the synthesized results.	4
Reporting bias assessment	14	Describe any methods used to assess risk of bias due to missing results in a synthesis (arising from reporting biases).	4
Certainty assessment	15	Describe any methods used to assess certainty (or confidence) in the body of evidence for an outcome.	4
**RESULTS**			
Study selection	16a	Describe the results of the search and selection process, from the number of records identified in the search to the number of studies included in the review, ideally using a flow diagram.	10
	16b	Cite studies that might appear to meet the inclusion criteria, but which were excluded, and explain why they were excluded.	10

Study characteristics	17	Cite each included study and present its characteristics.	10, 11
Risk of bias in studies	18	Present assessments of risk of bias for each included study.	11
Results of individual studies	19	For all outcomes, present, for each study: (a) summary statistics for each group (where appropriate) and (b) an effect estimate and its precision (e.g. confidence/credible interval), ideally using structured tables or plots.	11-16
Results of syntheses	20a	For each synthesis, briefly summarise the characteristics and risk of bias among contributing studies.	11-16
	20b	Present results of all statistical syntheses conducted. If meta-analysis was done, present for each the summary estimate and its precision (e.g. confidence/credible interval) and measures of statistical heterogeneity. If comparing groups, describe the direction of the effect.	16
	20c	Present results of all investigations of possible causes of heterogeneity among study results.	11-16
	20d	Present results of all sensitivity analyses conducted to assess the robustness of the synthesized results.	16-17
Reporting biases	21	Present assessments of risk of bias due to missing results (arising from reporting biases) for each synthesis assessed.	17
Certainty of evidence	22	Present assessments of certainty (or confidence) in the body of evidence for each outcome assessed.	11-16
**DISCUSSION**			
Discussion	23a	Provide a general interpretation of the results in the context of other evidence.	17
	23b	Discuss any limitations of the evidence included in the review.	17-18
	23c	Discuss any limitations of the review processes used.	17-18
	23d	Discuss implications of the results for practice, policy, and future research.	19
**OTHER INFORMATION**			
Registration and protocol	24a	Provide registration information for the review, including register name and registration number, or state that the review was not registered.	2
	24b	Indicate where the review protocol can be accessed, or state that a protocol was not prepared.	2
	24c	Describe and explain any amendments to information provided at registration or in the protocol.	2
Support	25	Describe sources of financial or	20

		non-financial support for the review, and the role of the funders or sponsors in the review.	
Competing interests	26	Declare any competing interests of review authors.	20
Availability of data, code and other materials	27	Report which of the following are publicly available and where they can be found: template data collection forms; data extracted from included studies; data used for all analyses; analytic code; any other materials used in the review.	20

Table S2. MOOSE Checklist for this systematic review and meta-analysis.

Criteria		Brief description of how the criteria were handled in the meta-analysis
**Reporting of background should include**		
√	Problem definition	Although there is clear evidence supporting the connection between breastfeeding and improved medical outcomes, debates have arisen regarding its correlation with NDOs.
√	Hypothesis statement	Diabetes increases the risk of active tuberculosis.
√	Description of study outcomes	Active tuberculosis disease
√	Type of exposure or intervention used	Breastfeeding
√	Type of study designs used	We included observation studies and RCTs.
√	Study population	Participates were full term infants.
**Reporting of search strategy should include**		
√	Qualifications of searchers	The credentials of the two investigators R.Z. and J.C. are indicated in the author list.
√	Search strategy, including time period included in the synthesis and keywords	The Cochrane Library, EMbase, Web of Science, CNKI, VIP, CBM, and WanFang Data were performed on December 17, 2023 from January 1, 2000.<br>See Table S3. in the article.
√	Databases and registries searched	PubMed, The Cochrane Library, EMbase, Web of Science, CNKI, VIP, CBM, and WanFang Data
√	Search software used, name and version, including special features	We did not employ a search software. EndNote was used to merge retrieved citations and eliminate duplications.
√	Use of hand searching	We have hand-checked the reference lists of original publications and previous meta-analyses or reviews.
√	List of citations located and those excluded, including justifications	Details of the literature search process are outlined in the flow chart. The citation list is available upon request.
√	Method of addressing articles published in languages other than English	We limited to studies published in the English or Chinese language.

√	Method of handling abstracts and unpublished studies	Unpublished data, conference papers, editorials, theses, and patents were not included.
√	Description of any contact with authors	We contacted corresponding authors of studies that did not reported sufficient data in an effort to complete our data set.
**Reporting of methods should include**		
√	Description of relevance or appropriateness of studies assembled for assessing the hypothesis to be tested	Detailed inclusion and exclusion criteria were described in the methods section.
√	Rationale for the selection and coding of data	Data extracted from each of the studies were relevant to the first author's name; year of publication; country; assessment point; age range; number of participants; assessment method of outcome; and confounding factors of interest.
√	Assessment of confounding	Restricted the analysis to breastfeeding category
√	Assessment of study quality, including blinding of quality assessors; stratification or regression on possible predictors of study results	The Newcastle-Ottawa Scale (NOS) adapted for cohort studies was used by two investigators to assess the quality of the included articles.
√	Assessment of heterogeneity	Heterogeneity of the studies were explored within two types of study designs using Cochrane's Q test of heterogeneity and I^2 statistic that provides the relative amount of variance of the summary effect due to the between-study heterogeneity.
√	Description of statistical methods in sufficient detail to be replicated	Description of methods of meta-analyses, sensitivity analyses, subgroup analysis and assessment of publication bias are detailed in the methods.
√	Provision of appropriate tables and graphics	We included 1 flow chart,1 summary table, 1table of subgroup analysis, 3 forest plot of all studies, 1 table of sensitivity analyses.
**Reporting of results should include**		
√	Graph summarizing individual study estimates and overall estimate	Figure 2.-4.
√	Table giving descriptive information for each study included	Table 1.
√	Results of sensitivity testing	Table S5.
√	Indication of statistical uncertainty of findings	95% confidence intervals were presented with all summary estimates, I^2 values and results of sensitivity analyses.
**Reporting of discussion should include**		

√	Quantitative assessment of bias	Sensitivity analyses indicate heterogeneity in strengths of the association due to most common biases in observational studies.
√	Justification for exclusion	We excluded studies that had not reported for the breastfeeding as exposure.
√	Assessment of quality of included studies	We discussed the results of the sensitivity analyses, and potential reasons for the observed heterogeneit.
**Reporting of conclusions should include**		
√	Consideration of alternative explanations for observed results	We discussed that potential unmeasured confounders such as the categorisation of BM dose, the neurodevelopmental test tools, demographic, social, and economic factors may related to the risk of NDOs.
√	Generalization of the conclusions	The results of this systematic review and meta-analysis, aimed at examining the association between breastfeeding and NDOs in preterm infants, provide support for breastfeeding recommendations. We noted the lack of studies in China.
√	Guidelines for future research	We recommend future studies on the effect of breastfeeding and NODs in preterm infants, should adjusting the factors, such as demographic, social, economic factors to re-evaluate the BF/NDOs association in relation to preterm infants.
√	Disclosure of funding source	This work has been supported by the Shenzhen Science and Technology Innovation Commission Fund, grant number JCYJ20230807152302005.

Table S3. Record of database search strategies - run 17/12/23.

#	Pubmed search strategy	Results
# 1	("Infant, Newborn"[Mesh]) OR (newborn OR neonate OR neonatal OR premature OR low birth weight OR VLBW OR LBW or infan* or neonat*)	1,850,665
# 2	((((breastmilk[tiab] OR (human[tiab] OR breast[tiab] OR mother*[tiab] OR maternal[tiab] OR express*[tiab] OR donor*[tiab] OR donated[tiab] OR bank*[tiab])) AND milk*[tiab])) OR ((breastfeed*[tiab] OR breastfed[tiab] OR ((breast[tiab] OR HM[tiab]) AND (fed[tiab] OR feed*[tiab]))))) OR(((EHM[tiab] OR MOM[tiab] OR PDM[tiab]) AND milk[tiab]))	106,048
# 3	("neurodevelopment disabilities"[tiab] OR "neurodevelopment outcome"[tiab] OR "neurodevelopment impairment" [tiab] OR "development outcome"[tiab] OR "development disorders" [tiab] OR "development disabilities"[tiab] OR "cognitive outcome"[tiab] OR "cognitive function"[tiab] OR "cognitive impairment"[tiab] OR "motor function"[tiab] OR "motor	392,991

	impairment"[tiab] OR "motor disorder"[tiab] OR "motor outcome"[tiab] OR "motor development" [tiab] OR "neuromotor development"[tiab] OR "neuromotor outcome" [tiab] OR "neuromotor impairment" [tiab] OR "neuromotor disorder"[tiab] OR "cerebral palsy" [tiab]) OR ("Developmental disabilities"[Mesh]OR "psychomotor disorders" [Mesh] OR "cognition disorders" [Mesh] OR "intellectual disability" [Mesh] OR "cerebral palsy" [Mesh] OR "Motor Skills Disorders" [Mesh])	
# 4	#1 AND #2 AND #3	587
# 5	#4 AND "humans"[MeSH Terms]	526
# 6	#5 NOT (Comment[sb] OR Letter[ptyp] OR Review[ptyp] OR (systematic[sb] OR Case Reports[ptyp]))	366
#7	#6 AND ((English[Language]) OR (Chinese[Language]))	355
#8	#7 AND ("2000/01/01"[Date - Publication] : "2023/12/17"[Date - Publication])	310
**#**	**Embase strategy**	**Results**
# 1	'newborn*':ab,ti OR 'neonate':ab,ti OR 'neonatal':ab,ti OR 'infant*':ab,ti OR 'neonat*':ab,ti OR 'preterm':ab,ti OR 'premature':ab,ti OR 'very low birth weight':ab,ti OR 'vlbw':ab,ti OR 'lbw':ab,ti	1,177,077
# 2	'developmental disorder'/exp OR 'psychomotor retardation'/exp OR 'motor retardation'/exp OR 'developmental coordination disorder'/exp OR 'cerebral palsy'/exp OR 'motor performance'/exp OR 'mild cognitive impairment'/exp OR 'cognitive development'/exp	252,067
# 3	'neurodevelopment disabilities':ab,ti OR 'neurodevelopment outcome':ab,ti OR 'neurodevelopment impairment':ab,ti OR 'development outcome':ab,ti OR 'development disorders':ab,ti OR 'development disabilities':ab,ti OR 'cognitive outcome':ab,ti OR 'cognitive function':ab,ti OR 'cognitive impairment':ab,ti OR 'motor function':ab,ti OR 'motor impairment':ab,ti OR 'motor disorder':ab,ti OR 'motor outcome':ab,ti OR 'motor development':ab,ti OR 'neuromotor development':ab,ti OR 'neuromotor outcome':ab,ti OR 'neuromotor impairment':ab,ti OR 'neuromotor disorder':ab,ti OR 'cerebral palsy':ab,ti	273,180
# 4	#2 OR #3	426,349
# 5	#4 AND #1	36,931
# 6	('breastmilk':ab,ti OR 'human':ab,ti OR 'breast':ab,ti OR 'mother*':ab,ti OR 'maternal':ab,ti OR 'express*':ab,ti OR 'donor*':ab,ti OR 'donated':ab,ti OR 'bank*':ab,ti) AND 'milk*':ab,ti	68,364
# 7	'breastfeed*':ab,ti OR 'breastfed':ab,ti OR (('breast':ab,ti OR 'hm':ab,ti) AND ('fed':ab,ti OR 'feed*':ab,ti))	80,333
# 8	('ehm':ab,ti OR 'mom':ab,ti OR 'pdm':ab,ti) AND 'milk':ab,ti	336
# 9	#6 OR #7 OR #8	129,079
# 10	#5 AND #9	1,110
# 11	#10 AND ([article]/lim OR [article in press]/lim) AND ([chinese]/lim OR [english]/lim) AND [humans]/lim AND	487

	[01-01-2000]/sd NOT [17-12-2023]/sd	
	**Cochrane search strategy**	**Results**
# 1	(((low birthweight) OR (low birth weight) OR VLBW OR ELBW OR (Prematur*) OR Preterm OR (Pre-term)) NEAR/1 ((Infant*) OR (Neonat*) OR (newborn*)) OR (new-born*) OR (baby*) OR babies) OR (neonatal intensive care) OR NICU	32,315
# 2	(((breastmilk OR (human OR breast OR mother* OR maternal OR express* OR donor* OR donated OR bank*)) NEAR/2 milk*) OR (breastfeed* OR breastfed OR ((breast OR HM) NEAR/0 (fed OR feed*))) OR ((EHM OR MOM OR PDM) AND milk) OR (Enteral* NEAR/0 (nutrition* OR support OR feed*)))	12,901
# 3	((brain OR neurologic* OR cognitive* OR intellectual* OR motor OR psychomotor) NEAR/1 development*) OR ((infan* OR child*) NEAR/1 development*) OR ((neurodevelopment*) OR (neuro-development*) OR (neurocognitive*) OR (neurocognitive) OR (neurobehavior*) OR (neurobehaviour*) OR (neuro-behavior*) OR (neuro-behaviour*) OR (neuropsycholog*) OR (neuro-psycholog*) OR cognition OR intelligence OR (executive function))	67,398
# 4	(#1 AND #2 AND #3)with Publication Year from 2000 to 2023, in Trials	399
	**Web of Science search strategy**	**Results**
# 1	(AB=((breastmilk OR human OR breast OR mother* OR maternal OR express* OR donor* OR donated OR bank*))) AND AB=(milk*)	43,622
# 2	AB=(breastfeed* OR breastfed OR breast OR hm) AND AB=(fed OR feed)	21,162
# 3	AB=(ehm OR mom OR pdm) AND AB=(milk)	197
# 4	#1 OR #2 OR #3	58,866
# 5	ALL=(neurodevelopment disabilities) OR (neurodevelopment outcome) OR (neurodevelopment impairment) OR (development outcome) OR (development disorders) OR (development disabilities) OR (cognitive outcome) OR (cognitive function) OR (cognitive impairment) OR (motor function) OR (motor impairment) OR (motor disorder) OR (motor outcome) OR (motor development) OR (neuromotor development) OR (neuromotor outcome) OR (neuromotor impairment) OR (neuromotor disorder) OR (cerebral palsy)	1,332,328
# 6	#4 AND #5	3441
# 7	AB=((newborn OR neonate OR neonatal OR premature OR low birth weight OR VLBW OR LBW or infan* or neonat*))	519,564
# 8	#6 AND #7	2,420
#9	#8 NOT WC=(surgery OR pharmacology pharmacy OR engineering OR anesthesiology OR urology nephrology OR telecommunications OR computer science OR otorhinolaryngology OR oncology OR music or mathematics OR mathematical computational biology OR genetics heredity or biophysics)	2,280

#10	#9 AND English (Languages) and Article (Document Types)	1,774
#11	#10 NOT TI=(case report or review or protocol)	1,680
#12	#11 AND DOP=(2000-01-01/2023-12-17)	1,653
**China National Knowledge Infrastructure (CNKI)**		
(主题=早产 + 早产儿 + 低出生体重儿 + VLB)<br>AND<br>(篇关摘=母乳 + 母乳喂养)<br>AND<br>(篇关摘=神经发育 + 认知 + 运动 + 语言)		155
**Wanfang Database (Wangfang)**		
(题名或关键词=早产 OR 早产儿 OR 低出生体重儿 OR VLB)<br>AND<br>(题名或关键词=母乳 OR 母乳喂养)<br>AND<br>(题名或关键词=神经发育 OR 认知 OR 运动 OR 语言)		42
**China Science and Technology Journal Database (VIP)**		
(题名或关键词=早产 + 早产儿 + 低出生体重儿 + VLB)<br>AND<br>(题名或关键词=母乳 + 母乳喂养)<br>AND<br>(题名或关键词=神经发育 + 认知 + 运动 + 语言)		52
**China Biology Medicine disc (CMB)**		
("早产"[标题:智能] OR "早产儿"[标题:智能] OR "低出生体重儿"[标题:智能] OR "VLB"[标题:智能])<br>AND<br>("母乳"[摘要:智能] OR "母乳喂养"[摘要:智能])<br>AND<br>("神经发育"[摘要:智能] OR "认知"[摘要:智能] OR "运动"[摘要:智能] OR "语言"[摘要:智能])		92

Table S4. Risk of bias of cohort studies.

	Selection				Comparability	Outcome			
**References**	**1**	**2**	**3**	**4**	**5**	**6**	**7**	**8**	**TOTAL**
Belfort et al. (2016)	*	*	*	*	**	*	*	*	9
Bier et al. (2002)	-	*	*	*	**	*	-	*	7
Colacci et al. (2017)	*	*	*	-	**	-	*	*	7
Feldman et al. (2003)	*	*	*	*	*-	*	-	*	7
Furman et al. (2004)	*	*	*	*	**	*	*	*	9
Hair et al. (2022)	*	*	*	-	**	-	*	*	7
Jacobi-Polishook et al. (2016)	*	*	*	*	**	*	-	*	8
Madore et al. (2017)	*	*	*	*	*-	*	-	-	6
O'Connor et al. (2003)	*	*	*	-	**	-	-	*	6
Patra et al. (2017)	*	*	*	-	**	-	*	*	7
Pinelli et al. (2003)	*	*	*	*	**	-	-	*	7
Rozéet al. (2012）	*	*	*	*	**	*	-	*	8
Tanaka et al. (2009)	-	*	*	*	*-	*	*	-	6
Vohr et al. (2006)	*	*	*	*	**	*	*	*	9
Were et al. (2006)	*	*	*	*	*-	*	*	-	7
Yackobovitch-Gavan et al. (2023)	*	*	*	-	**	-	-	*	6

Footnote: Not Reported: (-); Question 7= Minimum 18 months of follow-up; Question 8= Prospective cohort study and with the minimum 80% of follow up rate. 1）Representativeness of the exposed cohort; 2) Selection of the non exposed cohort; 3) Ascertainment of exposure; 4) Demonstration that outcome of interest was not present at start of study; 5) Comparability of cohorts on the basis of the design or analysis; 6) Assessment of outcome; 7) Whether the follow-up long enough for outcomes to occur; 8) Adequacy of follow up of cohorts.

Table S5. Sensitivity analyses by removing studies one by one for the pooled estimations.

**Never-BF vs. Any-BF: Cognitive Scores**			
**Author**	**ES**	**LL**	**UL**
Bier et al. (2002)	-0.1733651	-0.29571056	-0.05101964
Furman et al. (2004)	-0.20246303	-0.32813686	-0.07678922
Jacobi-Polishook et al. (2016)	-0.23172796	-0.363538	-0.09991793
Vohr et al. (2006)	-0.06499118	-0.29932776	0.16934538
**Never-BF vs. Any-BF: Motor Scores**			
**Author**	**ES**	**LL**	**UL**
Bier et al. (2002)	-.05852119	-0.44995242	0.33291006
Furmanet al. (2004)	-.24653894	-0.43609598	-0.0569819
Vohr et al. (2006)	-0.1639546	-0.82879275	0.50088358
Yackobovitch-Gavan et al. (2023)	-0.08002616	-0.58953834	0.42948601
**Pre-PTF vs. Pre-BF: Cognitive Scores**			
**Author**	**ES**	**LL**	**UL**
Colacci et al. (2017)	0.19108616	-0.00862812	0.39080042
Feldman et al. (2003)	0.14417921	-0.05456707	0.34292549
Furman et al. (2004)	0.14251776	-0.05571312	0.34074867
Hair et al. (2022)	0.11723977	-0.06785107	0.30233061
**Jacobi-Polishook et al. (2016)**	0.24191411	0.09145816	0.39237005
**Madore et al. (2017)**	0.20078751	0.01013905	0.39143598
O'Connor et al. (2003)	0.16340776	-0.06418473	0.39100024
Patra et al. (2017)	0.14370351	-0.06621323	0.35362023
Pinelli et al. (2003)	0.17780259	-0.03196939	0.38757458
Tanaka et al. (2009)	0.15670243	-0.03802066	0.3514255
**Pre-PTF vs. Pre-BF:Language Scores**			
**Author**	**ES**	**LL**	**UL**
Colacci et al. (2017)	0.2937119	-0.05139028	0.63881409
Hair et al. (2022)	0.24508397	-0.20979059	0.69995856
**Madore et al. (2017)**	**0.34421065**	**0.06807959**	**0.62034172**
Patra et al. (2017)	0.16448554	-0.04974515	0.37871623
**Pre-PTF vs. Pre-BF:Motor Scores**			
**Author**	**ES**	**LL**	**UL**
Colacci et al. (2017)	0.11065036	-0.06243343	0.28373414
Feldman et al. (2003)	0.06234159	-0.08331843	0.2080016
Furman et al. (2004)	0.13408232	-0.04011454	0.30827916
Hair et al. (2022)	0.12309258	-0.06544326	0.3116284
Jacobi-Polishook et al. (2016)	0.15752654	-0.03351514	0.34856823
Madore et al. (2017)	0.12282848	-0.04920088	0.29485786
O'Connor et al. (2003)	0.1601564	-0.01977779	0.34009057
Patra et al. (2017)	0.08079701	-0.08868451	0.25027853
Pinelli et al. (2003)	0.12513517	-0.055594	0.30586433
Tanaka et al. (2009)	0.07595058	-0.06465971	0.21656087

Footnote: The effect size and 95% interval confidence, represent the pooled estimations after excluding from the analysis the corresponding reference.

Abbreviations: BF, Breastfeeding; Pre-, Predominant; PTF, Preterm Formula; ES, Effect Size; LL, Low Limit, UL, Upper Limit. NDOs, Neurodevelopment Outcomes.

Table S6. Subgroup analyses of the relationship between BF and NDOs.

SUBGROUP	Never-BF vs. Any-BF: Motor Scores	No. of studies	SMD [95%CI]	I^2	P value
Assessment Points	<18 months	1	-0.38 [-0.79, 0.03]	-	0.07
	18 months to 24 months	2	-0.06 [-0.45, 0.33]	77	0.77
	>24 months	0	-	-	-
GA and BW	< 32 wk. and/or <1,500 g	2	0.03 [-0.79, 0.85]	87	0.94
	< 28 wk. and/or <1,000 g	1	-0.18 [-0.32, -0.04]	-	0.01
Maternal education/IQ	Adjusted	5	-0.08 [-0.37, 0.21]	71	0.60
	Unadjusted	0	-	-	-
	**Pre-PTF vs. Pre-BF: Cognitive Scores**	**No. of studies**	**SMD [95%CI]**	I^2	***P* value**
Assessment Points	<18 months	3	0.19 [0.03, 0.35]	0	0.02
	18 months to 24 months	6	0.12 [-0.17, 0.42]	80	0.42
	>24 months	1	0.45 [-0.49, 1.40]	-	0.35
GA and BW	< 32 wk. and/or <1,500g	6	0.21 [-0.02, 0.43]	53	0.08
	< 28 wk. and/or <1,000g	2	-0.24 [-0.65, 0.16]	0	0.24
Maternal education/IQ	Adjusted	6	0.15 [-0.07, 0.38]	71	0.18
	Unadjusted	4	0.17 [-0.21, 0.55]	62	0.37
	**Pre-PTF vs. Pre-BF: Language Scores**	**No. of studies**	**SMD [95%CI]**	I^2	***P* value**
Assessment Points	<18 months	-	-	-	-
	18 months to 24 months	4	0.27 [-0.01, 0.55]	57	0.06
	>24 months	-	-	-	-
GA and BW	< 32 wk. and/or <1,500g	4	0.27 [-0.01, 0.55]	57	0.06
	< 28 wk. and/or <1,000g	2	0.00 [-0.40, 0.41]	0	0.99
Maternal education/IQ	Adjusted	1	0.12 [-0.40, 0.64]	-	0.66
	Unadjusted	3	0.29 [-0.05, 0.64]	68	0.1
	**Pre-PTF vs. Pre-BF: Motor Scores**	**No. of studies**	**SMD [95%CI]**	I^2	***P* value**
Assessment Points	<18 months	3	0.07 [-0.29, 0.43]	76	0.47
	18 months to 24 months	6	0.05 [-0.06, 0.17]	18	0.35
	>24 months	1	1.37 [0.31, 2.42]	-	0.01
GA and BW	< 32 wk. and/or <1,500g	6	0.14 [0.00, 0.29]	0	0.05
	< 28 wk. and/or <1,000g	2	0.12 [-0.28, 0.52]	0	0.56
Maternal education/IQ	Adjusted	7	0.16 [-0.08, 0.39]	70	0.19
	Unadjusted	3	0.09 [-0.11, 0.28]	0	0.38

Abbreviations: BF, Breastfeeding; Pre-, Predominant; PTF, Preterm Formula; NDOs, Neurodevelopment Outcomes; No., Number; wk., Weeks

Table S7. Summary of findings.

Outcomes	Anticipated absolute effects* (95% CI)		Relative effect (95% CI)	No. of participants (studies)	Certainty of the evidence (GRADE)
	Risk with Non breastfeeding	Risk with breastfeeding			
**Cognitive Scores**					
Never-BF vs. Any-BF	The mean cognitive scores in the Never-BF groups was 0.19 standard deviations lower (0.31 to 0.07 lower)	-	-	1783 (4 observation studies)	⊕◯◯◯ Very low[1]
Pre-PTF vs. Pre-BF	The mean cognitive scores in the Pre-PTF groups was 0.17 standard deviations higher (0.02 lower to 0.36 higher)	-	-	1903 (10 observation studies)	⊕◯◯◯ Very low[2]
Pre-PTF vs. Pre-DBM: Cohort Studies	The mean cognitive scores-cohort studies in the Pre-PTF was 0.51 standard deviations higher (0.26 to 0.75 higher)	-	-	281 (2 observation studies)	⊕ ⊕◯◯ Low
Pre-PTF vs. Pre-DBM: RCTs	The mean cognitive scores-RCTs in the Pre-PTF groups was 0.08 standard deviations higher (0.14 lower to 0.31 higher)	-	-	299 (1 RCT)	⊕ ⊕ ⊕◯ Moderate[4]
**Language Scores**					
Pre-PTF vs. Pre-BF	The mean language scores in the Pre-PTF groups was 0.27 standard deviations higher (0.01 lower to 0.55 higher)	-	-	605 (4 observation studies)	⊕◯◯◯ Very low [2]
Pre-PTF vs. Pre-DBM: Cohort Studies	The mean language scores-cohort studies in the Pre-PTF groups was 0.17 standard deviations higher (0.06 lower to 0.41 higher)	-	-	299 (2 observation studies)	⊕ ⊕◯◯ Low
Pre-PTF vs. Pre-DBM: RCTs	The mean language scores-RCTs studies groups was 0.14 standard deviations higher (0.09 lower to 0.36 higher)	-	-	299 (1 RCT)	⊕ ⊕ ⊕◯ Moderate[4]

**Motor Scores**					
Never-BF vs. Any-BF	The mean motor scores in the Never-BF groups was 0.15 standard deviations lower (0.51 lower to 0.21 higher)	-	-	1303 (4 observation studies)	⊕◯◯◯ Very low[1,2]
Pre-PTF vs. Pre-BF	The mean motor scores in the Pre-PTF groups was 0.11 standard deviations higher (0.05 lower to 0.28 higher)	-	-	1900 (10 observation studies)	⊕◯◯◯ Very low [2,5]
Pre-PTF vs. Pre-DBM: Cohort Studies	The mean motor scores -cohort studies in the Pre-PTF groups was 0.1 standard deviations higher (0.14 lower to 0.33 higher)	-	-	299 (2 observation studies)	⊕ ⊕◯◯ Low
Pre-PTF vs. Pre-DBM: RCTs	TThe mean motor scores-RCTs studies in the Pre-PTF groups was 0.12 standard deviations higher (0.11 lower to 0.34 higher)	-	-	299 (1 RCT)	⊕ ⊕ ⊕◯ Moderate[4]
**Incidence of NDI**	**Study population**				
Never-BF vs. Any-BF	571 per 1000	344 per 1000	RR 1.17 (1.07 to 1.29)	1133 (2 observation studies)	⊕◯◯◯ Very low [1,3]
Pre-PTF vs. Pre-BF	71 per 1000	73 per 1000 (13 to 410)	RR 1.02 (0.18 to 5.74)	69 (1 observation studies)	⊕◯◯◯ Very low[4]
Pre-PTF vs. Pre-DBM: RCTs	272 per 1000	163 per 1000 (103 to 255)	RR 0.6 (0.38 to 0.94)	299 (1 RCT)	⊕ ⊕ ⊕◯ Moderate[4]

CI: Confidence interval; RR: Risk ratio; BF, Breastfeeding; Pre-, Predominant; PTF, Preterm Formula; NDOs, Neurodevelopment Outcomes; GRADE Working Group grades of evidence

High quality: Further research is very unlikely to change our confidence in the estimate of effect.

Moderate quality: Further research is likely to have an important impact on our confidence in the estimate of effect and may change the estimate.

Low quality: Further research is very likely to have an important impact on our confidence in the estimate of effect and is likely to change the estimate.

Very low quality: We are very uncertain about the estimate.

[1] 1 study contributes a large study population;
[2] Moderate heterogeneity;
[3] Small sample size;
[4] Only 1 study;
[5] Egger test: p=0.048.

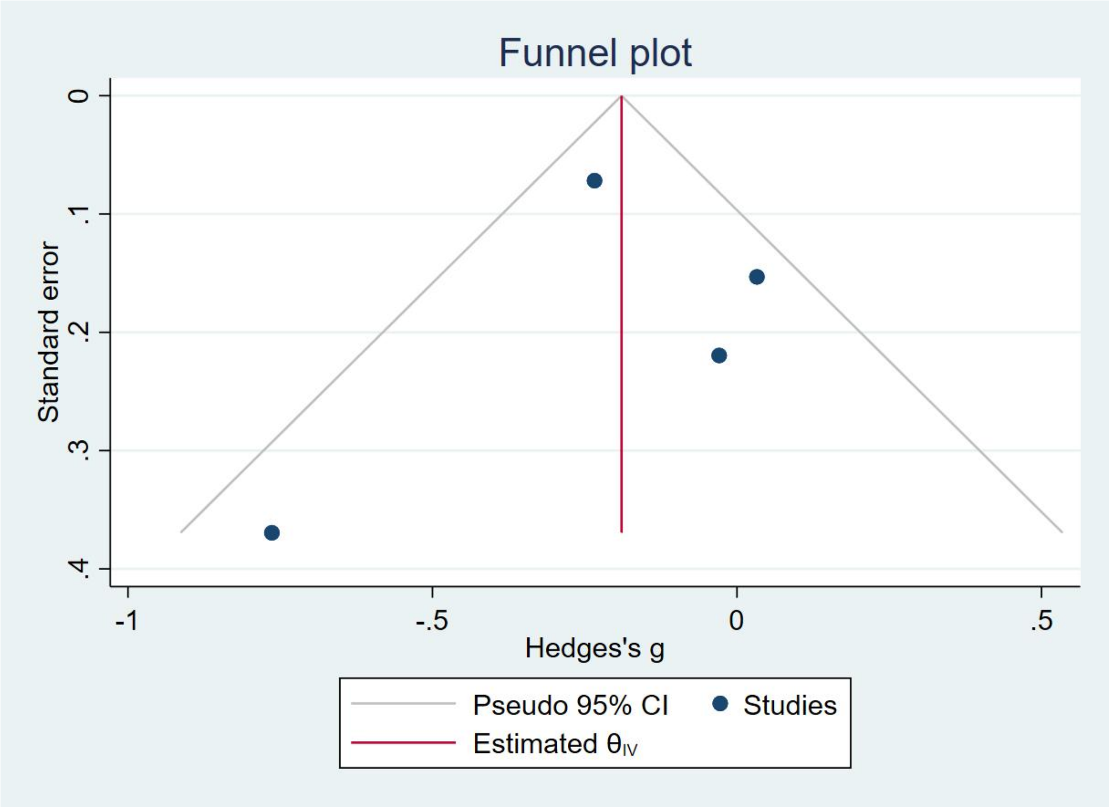

Figure S1. Funnel plot for publication bias using Egger test, for the association between Never-BF vs. Any-BF on cognitive scores. Coef. 0.0228846, $p = 0.990$.

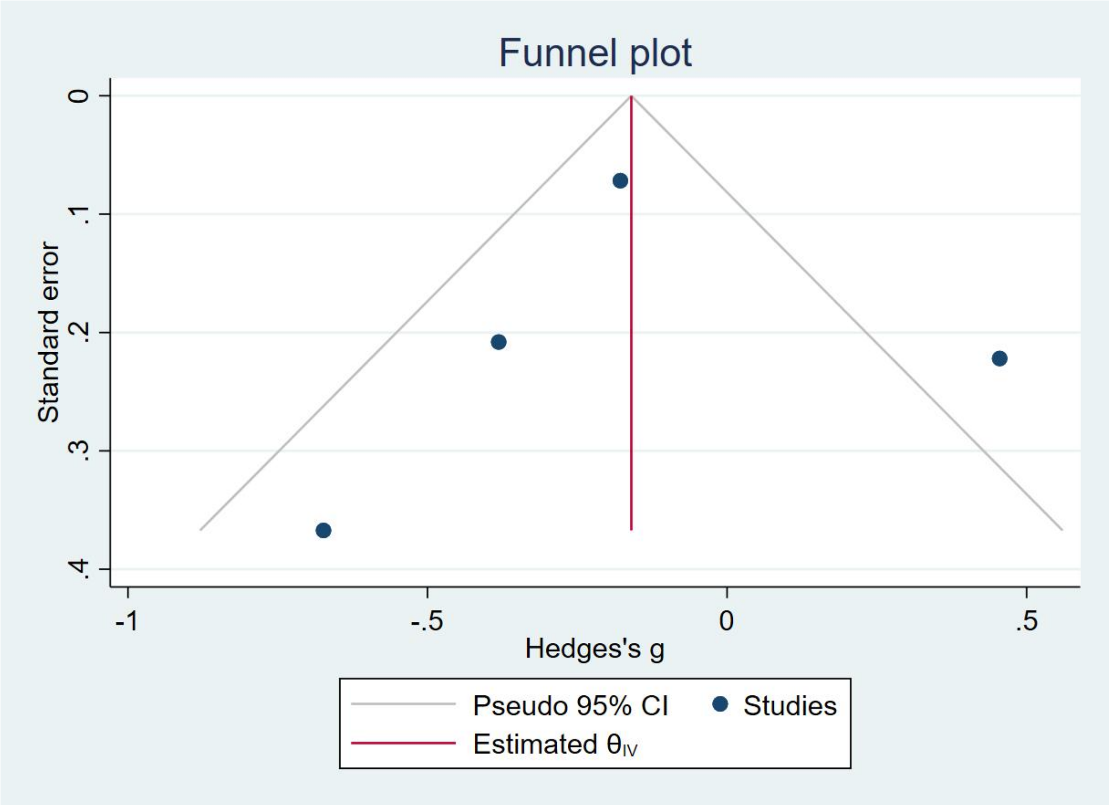

Figure S2. Funnel plot for publication bias using Egger test, for the association between Never-BF vs. Any-BF on motor scores. Coef. 0.0501772, $p = 0.983$.

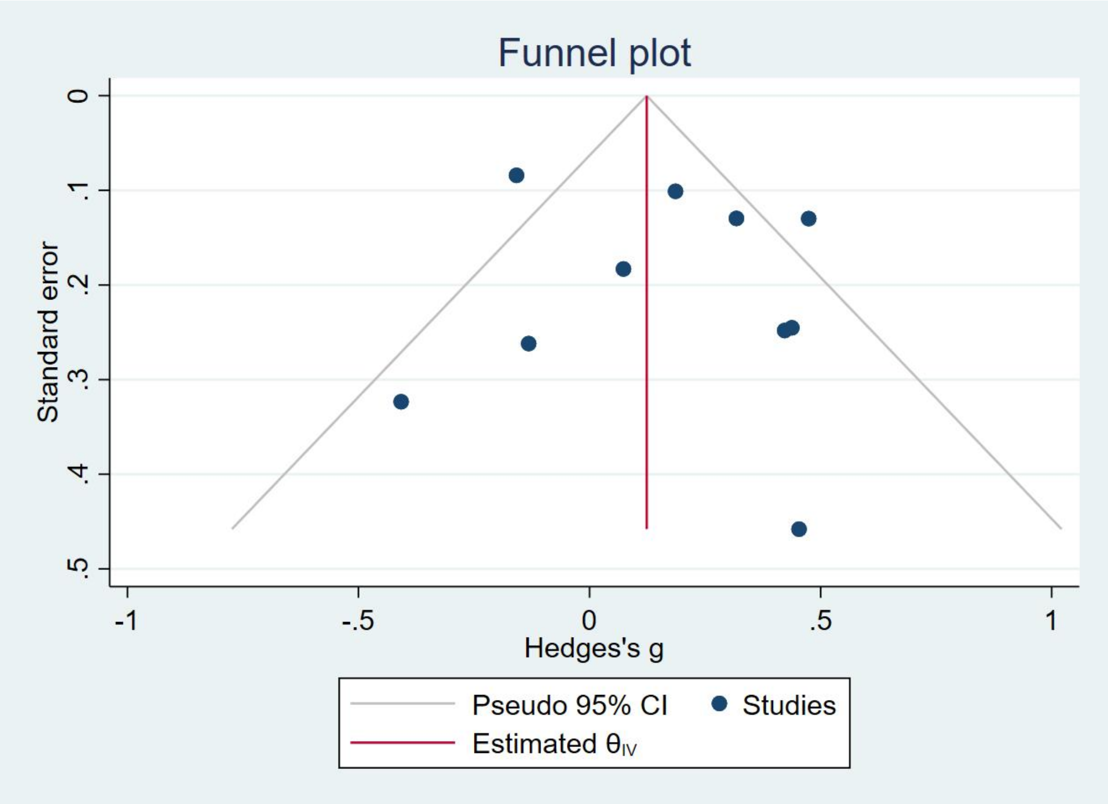

Figure S3. Funnel plot for publication bias using Egger test, for the association between Pre-PTF vs. Pre-BF on cognitive scores. Coef. 0.8535165, p=0.519.

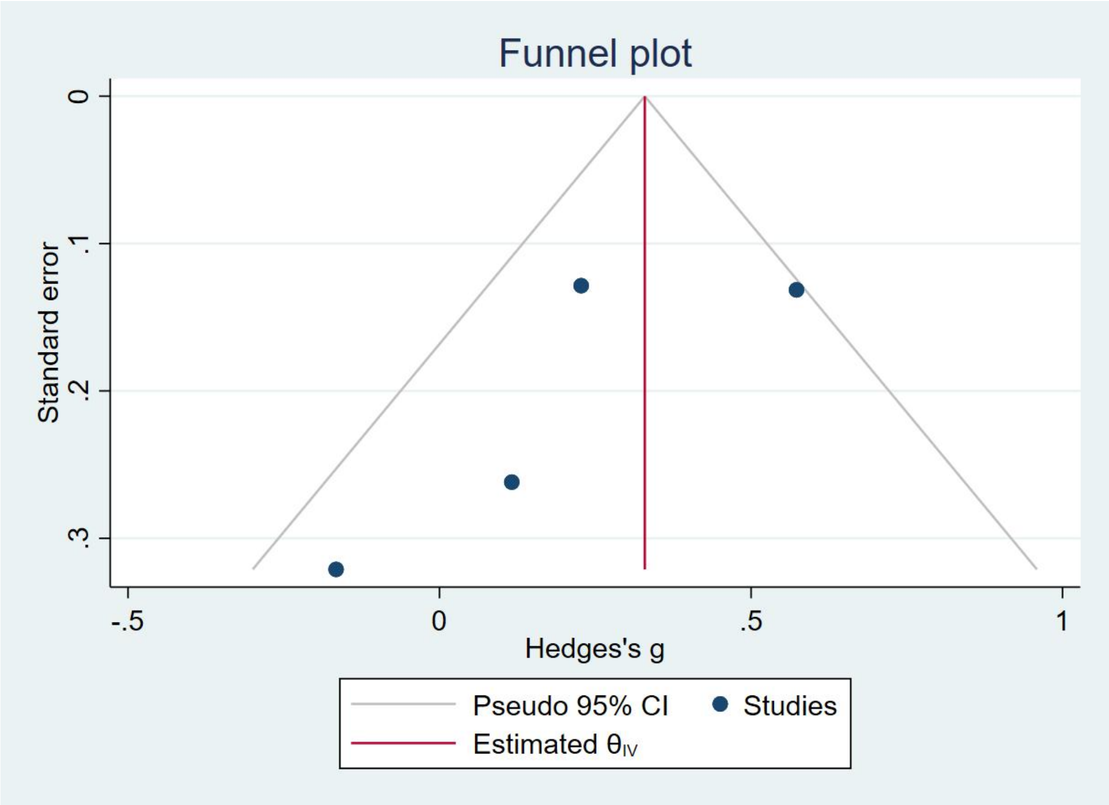

Figure S4. Funnel plot for publication bias using Egger test, for the association between Pre-PTF vs. Pre-BF on language scores. Coef. -2.491389, p=0.320.

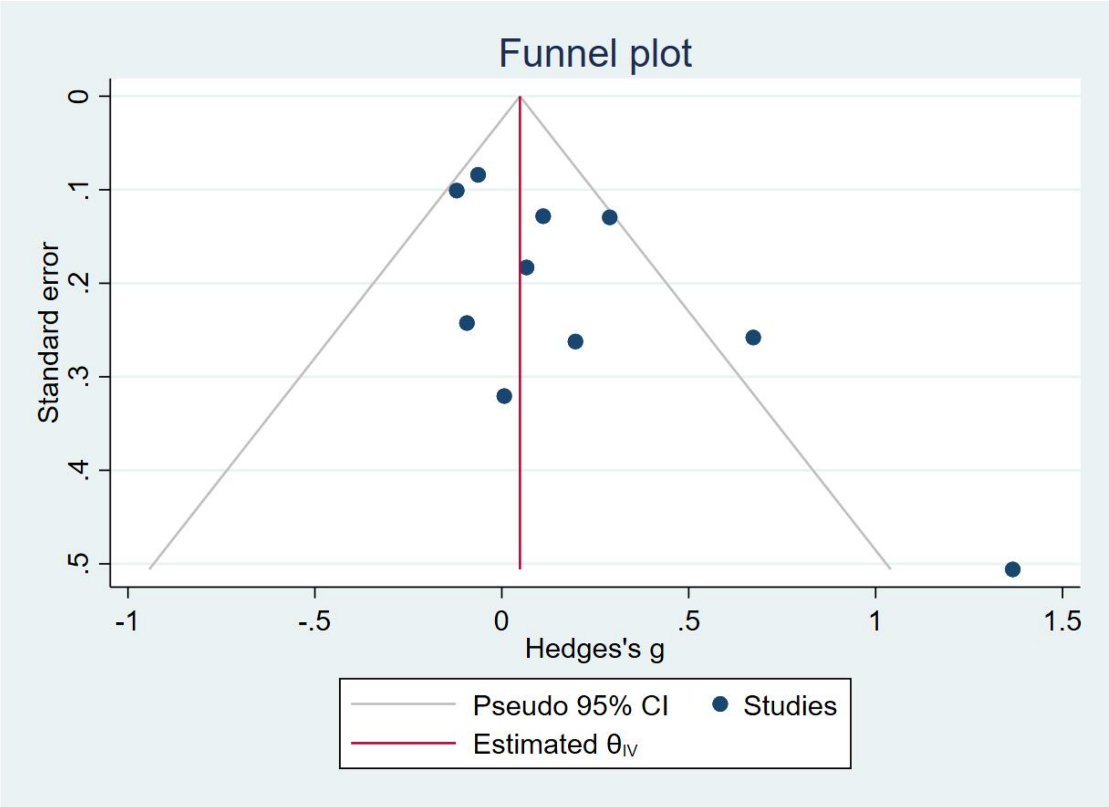

Figure S5. Funnel plot for publication bias using Egger test, for the association between Pre-PTF vs. Pre-BF on motor scores. Coef. 1.975435, *p*=0.048

Excluded references.(1-41)

1. Agostoni C, Marangoni F, Lammardo AM, Giovannini M, Riva E, Galli C. Breastfeeding duration, milk fat composition and developmental indices at 1 year of life among breastfed infants. Prostaglandins Leukot Essent Fatty Acids. 2001;64(2):105-9.doi:10.1054/plef.2001.0248

2. Bajwa RU, Raju MNP, Govande VP, Hemingway M, Hammonds K, Vora N. Infant nutrition (donor human milk vs. maternal milk) and long-term neurodevelopmental and growth outcomes in very low birth weight infants. J Matern-Fetal Neonatal Med. 2022;35(25):10025-9.doi:10.1080/14767058.2022.2086794

3. Beaino G, Khoshnood B, Kaminski M, Marret S, Pierrat V, Vieux R, et al. Predictors of the risk of cognitive deficiency in very preterm infants: the EPIPAGE prospective cohort. ACTA PAEDIATRICA. 2011;100(3):370-8.doi:10.1111/j.1651-2227.2010.02064.x %\ 2024-01-03 16:12:00

4. Belfort MB. Human Milk and Preterm Infant Brain Development. BREASTFEEDING MEDICINE. 2018;13(S1):S23-S5.doi:10.1089/bfm.2018.29079.mbb

5. Bellando J, McCorkle G, Spray B, Sims CR, Badger TM, Casey PH, et al. Developmental assessments during the first 5 years of life in infants fed breast milk, cow's milk formula, or soy formula. FOOD SCIENCE & NUTRITION. 2020;8(7):3469-78.doi:10.1002/fsn3.1630 %\ 2024-01-03 22:02:00

6. Bergner EM, Shypailo R, Visuthranukul C, Hagan J, O'Donnell AR, Hawthorne KM, et al. Growth, Body Composition, and Neurodevelopmental Outcomes at 2 Years Among Preterm Infants Fed an Exclusive Human Milk Diet in the Neonatal Intensive Care Unit: A Pilot Study. BREASTFEEDING MEDICINE. 2020;15(5):304-11.doi:10.1089/bfm.2019.0210 %\ 2024-02-28 15:40:00

7. Chiu WC, Liao HF, Chang PJ, Chen PC, Chen YC. Duration of breast feeding and risk of developmental delay in Taiwanese children: a nationwide birth cohort study. PAEDIATRIC AND PERINATAL EPIDEMIOLOGY. 2011;25(6):519-27.doi:10.1111/j.1365-3016.2011.01236.x %\ 2024-02-06 10:02:00

8. Choi HJ, Kang SK, Chung MR. The relationship between exclusive breastfeeding and infant development: A 6- and 12-month follow-up study. Early Hum Dev. 2018;127:42-7.doi:10.1016/j.earlhumdev.2018.08.011

9. Collins CT, Gibson RA, Anderson PJ, McPhee AJ, Sullivan TR, Gould JF, et al. Neurodevelopmental outcomes at 7 years' corrected age in preterm infants who were fed high-dose docosahexaenoic acid to term equivalent: a follow-up of a randomised controlled trial. BMJ OPEN. 2015;5(3):e007314.doi:10.1136/bmjopen-2014-007314 %/ Published by the BMJ Publishing Group Limited. For permission to use (where not already granted under a licence) please go to http://group.bmj.com/group/rights-licensing/permissions.

10. Corpeleijn WE, de Waard M, Christmann V, van Goudoever JB, Jansen-van Der Weide MC, Kooi E, et al. Effect of Donor Milk on Severe Infections and Mortality in Very Low-Birth-Weight Infants The Early Nutrition Study Randomized Clinical Trial. JAMA PEDIATRICS. 2016;170(7):654-61.doi:10.1001/jamapediatrics.2016.0183 %\ 2024-01-06 11:12:00

11. Dewey KG, Cohen RJ, Brown KH, Rivera LL. Effects of exclusive breastfeeding for four versus six months on maternal nutritional status and infant motor development: results of two randomized trials in Honduras. 2001;131(2):262-7.doi:10.1093/jn/131.2.262

12. Drane DL, Logemann JA. A critical evaluation of the evidence on the association between type of infant feeding and cognitive development. PAEDIATRIC AND PERINATAL EPIDEMIOLOGY. 2000;14(4):349-56.doi:10.1046/j.1365-3016.2000.00301.x %\ 2024-02-06 09:55:00
13. Eickmann SH, Malkes NF, Lima MC. Psychomotor development of preterm infants aged 6 to 12 months. Sao Paulo Med J. 2012;130(5):299-306.doi:10.1590/s1516-31802012000500006
14. Eidelman AI, Feldman R. Positive effect of human milk on neurobehavioral and cognitive development of premature infants. In: Pickering LK, Morrow AL, RuizPalacios GM, Schanler RJ, editors. 554. 11th International Conference of the International-Society-for-Research-in-Human-Milk-and-Lactation2004. p. 359-64.
15. Elgen I, Sommerfelt K, Ellertsen B. Cognitive performance in a low birth weight cohort at 5 and 11 years of age. PEDIATRIC NEUROLOGY. 2003;29(2):111-6.doi:10.1016/s0887-8994(03)00211-x %\ 2023-12-25 10:23:00
16. Ericson J, Ahlsson F, Wackernagel D, Wilson E. Equally Good Neurological, Growth, and Health Outcomes up to 6 Years of Age in Moderately Preterm Infants Who Received Exclusive vs. Fortified Breast Milk-A Longitudinal Cohort Study. NUTRIENTS. 2023;15(10).doi:10.3390/nu15102318 %\ 2024-01-23 09:56:00
17. Fewtrell MS, Morley R, Abbott RA, Singhal A, Isaacs EB, Stephenson T, et al. Double-blind, randomized trial of long-chain polyunsaturated fatty acid supplementation in formula fed to preterm infants. 2002;110(1):73-82
18. Girard LC, Farkas C. Breastfeeding and behavioural problems: Propensity score matching with a national cohort of infants in Chile. BMJ OPEN. 2019;9(2).doi:10.1136/bmjopen-2018-025058 %\ 2024-02-06 10:09:00
19. Gsollpointner M, Eibensteiner F, Thanhaeuser M, Fuiko R, Jilma B, Ristl R, et al. POLYUNSATURATED FATTY ACID INTAKE DURING COMPLEMENTARY FEEDING AND NEUROLOGICAL DEVELOPMENT IN VERY LOW BIRTH WEIGHT INFANTS: a SECONDARY ANALYSIS OF A RANDOMIZED INTERVENTION TRIAL. 2023;76:1028-9.doi:10.1097/mpg.0000000000003823
20. Hopperton KE, OConnor DL, Bando N, Conway AM, Ng D, Kiss A, et al. Nutrient Enrichment of Human Milk with Human and Bovine Milk-Based Fortifiers for Infants Born <1250 g: 18-Month Neurodevelopment Follow-Up of a Randomized Clinical Trial. 2019;3(12):nzz129.doi:10.1093/cdn/nzz129
21. Horwood LJ, Darlow BA, Mogridge N. Breast milk feeding and cognitive ability at 7-8 years. ARCHIVES OF DISEASE IN CHILDHOOD-FETAL AND NEONATAL EDITION. 2001;84(1):F23-F7.doi:10.1136/fn.84.1.F23 %\ 2024-02-06 09:55:00
22. Isaacs EB, Fischl BR, Quinn BT, Chong WK, Gadian DG, Lucas A. Impact of Breast Milk on Intelligence Quotient, Brain Size, and White Matter Development. PEDIATRIC RESEARCH. 2010;67(4):357-62.doi:10.1203/PDR.0b013e3181d026da %\ 2024-02-06 09:49:00
23. Johnson S, Wolke D, Hennessy E, Marlow N. Educational Outcomes in Extremely Preterm Children: Neuropsychological Correlates and Predictors of Attainment. DEVELOPMENTAL NEUROPSYCHOLOGY. 2011;36(1):74-95.doi:10.1080/87565641.2011.540541 %\ 2024-02-06 10:02:00
24. Keim SA, Sullivan JA, Sheppard K, Smith K, Ingol T, Boone KM, et al. Feeding Infants at the Breast or Feeding Expressed Human Milk: Long-Term Cognitive, Executive Function, and Eating

Behavior Outcomes at Age 6 Years. JOURNAL OF PEDIATRICS. 2021;233:66-+.doi:10.1016/j.jpeds.2021.02.025 %\ 2024-01-09 22:11:00
25. Kim KM, Choi JW. Associations between breastfeeding and cognitive function in children from early childhood to school age: a prospective birth cohort study. Int Breastfeed J. 2020;15(1):83.doi:10.1186/s13006-020-00326-4
26. Klamer A, Toftlund LH, Grimsson K, Halken S, Zachariassen G. IQ Was Not Improved by Post-Discharge Fortification of Breastmilk in Very Preterm Infants. 2022;14(13).doi:10.3390/nu14132709
27. Lenehan SM, Boylan GB, Livingstone V, Fogarty L, Twomey DM, Nikolovski J, et al. The impact of short-term predominate breastfeeding on cognitive outcome at 5 years. ACTA PAEDIATRICA. 2020;109(5):982-8.doi:10.1111/apa.15014 %\ 2024-02-06 09:55:00
28. Leventakou V, Roumeliotaki T, Koutra K, Vassilaki M, Mantzouranis E, Bitsios P, et al. Breastfeeding duration and cognitive, language and motor development at 18 months of age: Rhea mother-child cohort in Crete, Greece. JOURNAL OF EPIDEMIOLOGY AND COMMUNITY HEALTH. 2015;69(3):232-9.doi:10.1136/jech-2013-202500 %\ 2023-12-25 16:26:00
29. Lucas A, Fewtrell MS, Morley R, Singhal A, Abbott RA, Isaacs E, et al. Randomized trial of nutrient-enriched formula versus standard formula for postdischarge preterm infants. PEDIATRICS. 2001;108(3):703-11.doi:10.1542/peds.108.3.703
30. McCrory C, Murray A. The Effect of Breastfeeding on Neuro-Development in Infancy. MATERNAL AND CHILD HEALTH JOURNAL. 2013;17(9):1680-8.doi:10.1007/s10995-012-1182-9 %\ 2024-01-18 21:40:00
31. Morley R, Lucas A. Randomized diet in the neonatal period and growth performance until 7.5-8 y of age in preterm children. Am J Clin Nutr. 2000;71(3):822-8.doi:10.1093/ajcn/71.3.822
32. Oddy WH, Kendall GE, Blair E, de Klerk NH, Stanley FJ, Landau LI, et al. Breast feeding and cognitive development in childhood: a prospective birth cohort study. PAEDIATRIC AND PERINATAL EPIDEMIOLOGY. 2003;17(1):81-90.doi:10.1046/j.1365-3016.2003.00464.x %\ 2023-12-25 16:38:00
33. Oddy WH, Robinson M, Kendall GE, Li J, Zubrick SR, Stanley FJ. Breastfeeding and early child development: a prospective cohort study. ACTA PAEDIATRICA. 2011;100(7):992-9.doi:10.1111/j.1651-2227.2011.02199.x %\ 2024-01-10 08:49:00
34. Pineda R, Muñoz R, Chrzastowski H, Dunsirn-Baillie S, Wallendorf M, Smith J. Maternal Milk and Relationships to Early Neurobehavioral Outcome in Preterm Infants. JOURNAL OF PERINATAL & NEONATAL NURSING. 2020;34(1):72-9.doi:10.1097/jpn.0000000000000460 %\ 2024-02-06 09:55:00
35. Rodrigues C, Zeitlin J, Zemlin M, Wilson E, Pedersen P, Barros H. Never-breastfed children face a higher risk of suboptimal cognition at 2 years of corrected age: A multinational cohort of very preterm children. Matern Child Nutr. 2022;18(3).doi:10.1111/mcn.13347
36. Rozé JC, Darmaun D, Boquien CY, Flamant C, Picaud JC, Savagner C, et al. The apparent breastfeeding paradox in very preterm infants: relationship between breast feeding, early weight gain and neurodevelopment based on results from two cohorts, EPIPAGE and LIFT. BMJ OPEN. 2012;2(2).doi:10.1136/bmjopen-2012-000834 %\ 2024-01-14 12:36:00
37. Ruys CA, Bröring T, van Schie PEM, van de Lagemaat M, Rotteveel J, Finken MJJ, et al. Neurodevelopment of children born very preterm and/or with a very low birth weight: 8-Year follow-up of a nutritional RCT. 2019;30:190-8

38. Smith MM, Durkin M, Hinton VJ, Bellinger D, Kuhn L. Influence of breastfeeding on cognitive outcomes at age 6-8 years: Follow-up of very low birth weight infants. AMERICAN JOURNAL OF EPIDEMIOLOGY. 2003;158(11):1075-82.doi:10.1093/aje/kwg257 %\ 2024-02-28 11:21:00
39. Stelmach I, Kwarta P, Jerzyńska J, Stelmach W, Krakowiak J, Karbownik M, et al. Duration of breastfeeding and psychomotor development in 1-year-old children - Polish Mother and Child Cohort Study. Int J Occup Med Environ Health. 2019;32(2):175-84.doi:10.13075/ijomeh.1896.01328 %/ This work is available in Open Access model and licensed under a CC BY-NC 3.0 PL license.
40. Vohr BR, Poindexter BB, Dusick AM, McKinley LT, Higgins RD, Langer JC, et al. Persistent beneficial effects of breast milk ingested in the neonatal intensive care unit on outcomes of extremely low birth weight infants at 30 months of age. 2007;120(4):e953-9.doi:10.1542/peds.2006-3227
41. Zhang Y, Deng QQ, Wang JH, Wang H, Li QF, Zhu BH, et al. The impact of breast milk feeding on early brain development in preterm infants in China: An observational study. PLOS ONE. 2022;17(11).doi:10.1371/journal.pone.0272125 %\ 2024-02-29 17:43:00
